# Patterns of opioid prescribing to opioid‐naive patients after surgical and emergency care: A population‐based cross‐sectional study using linked administrative databases in Nova Scotia (2017–2019)

**DOI:** 10.1111/dar.14029

**Published:** 2025-03-07

**Authors:** Roah A. Merdad, Mark Asbridge, Samuel Campbell, Daniel J. Dutton, Jill A. Hayden

**Affiliations:** ^1^ Department of Community Health and Epidemiology Dalhousie University Halifax Canada; ^2^ Department of Community Medicine King Abdulaziz University Jeddah Saudi Arabia; ^3^ Department of Emergency Medicine Dalhousie University Halifax Canada; ^4^ Emergency Medicine Nova Scotia Health Authority, QEII Health Sciences Centre Halifax Canada; ^5^ Horizon Health Network Saint John Canada

**Keywords:** emergency care, opioids, prescribing patterns, provider specialty, surgical care

## Abstract

**Introduction:**

To describe opioid prescribing patterns for opioid‐naive patients who filled prescriptions after surgical or emergency care.

**Methods:**

We conducted a population‐based, cross‐sectional study of opioid‐naive adults who filled opioid prescriptions within 14 days of receiving surgical or emergency care in Nova Scotia, Canada. Using linked administrative databases, we estimated the prevalence of opioid prescriptions with >7 days' supply, ≥90 morphine milligram equivalents (MME)/day or long‐acting opioids. We assessed the association of care setting and specialty with these outcomes.

**Results:**

Among 124,515 patients, 36,716 (29.5%) were opioid‐naive. The median opioid supply duration was 3 days (IQR 2–5), the median dose was 50 MME/day (IQR 30–75). Prescriptions for >7 days, ≥90 MME/day or involving long‐acting opioids were filled by 10.9%, 20.2% and 0.7% of the patients, respectively. Hydromorphone (50%) and codeine (26.4%) were the most filled opioids. The emergency care setting had double the odds of filling >7 days' supply (OR 2.13, 95% CI 1.99–2.28), and 69% lower chance of filling ≥90 MME/day (OR 0.31, 95% CI 0.29–0.33) than surgical care. In the surgical care setting, there was significant variation across medical specialties. Otolaryngology was associated with a higher chance of prescribing >7 days' opioid supply than general surgery (OR 4.89, 95% CI 3.86–6.20). Orthopaedic surgery had a higher likelihood of ≥90 MME/day prescriptions (OR 2.92, 95% CI 2.58–3.30) than general surgery.

**Discussion and Conclusions:**

Opioid prescribing patterns vary significantly by setting and specialty in Nova Scotia, Canada. Our results emphasise the need for tailored guidelines that consider clinical context and specialty to enhance patient safety and reduce opioid misuse risk.

## 1 INTRODUCTION

### 1.1 Background

Each year, thousands of patients in Canada interact with the healthcare system for acute pain requiring emergency care, or receive surgical care that is associated with significant pain postoperatively [1, 2, 3]. Opioids are frequently prescribed in these settings, with recent evidence finding that over three‐quarters of patients who had surgery between 2013 and 2016 in Canada filled opioids within 7 days of discharge [4], and over one‐third of patients who visited an emergency department in Nova Scotia for non‐specific low‐back pain between 2009 and 2015 were discharged with an opioid prescription [5]. Many patients who receive opioids for acute pain are opioid‐naive at the time [6, 7]. Opioid‐naivety is frequently defined as no documented opioid use in the 6 to 12 months preceding a new fill. In the year 2018, 8.1% of the Canadian population started opioids while opioid‐naïve [6].

Certain patterns of prescribing have been shown to be associated with higher risk of opioid‐related harms in opioid‐naive populations. Prescriptions that are longer in duration, higher in dose or for long‐acting opioids may be associated with higher risk of prolonged use [8, 9], misuse [10] and overdose [11]. Canadian quality monitoring initiatives for opioid prescribing to patients with acute pain, and opioid prescribing guidelines for post‐operative and injury‐related pain in the United States recommend that, when opioids are deemed necessary to treat acute pain in opioid‐naive populations, prescriptions are written for short‐acting formulations in the lowest effective dose for the shortest duration possible [12, 13, 14]. In general, these guidelines indicate that 3 days are often sufficient and exceeding 7 days' supply is rarely needed.

Although thresholds for prescribing are only meant to guide care for the majority of, but not all, patients, and first‐prescriptions that are written in excess of 1 week or in high‐dose may be necessary for some opioid‐naive patients for whom recovery duration is expected to be long or who are expected to have severe pain, evidence suggests that physicians frequently prescribe opioids in excess of patient need. A systematic review on quantity of opioids consumed after surgery, and a Canadian study that followed patients after emergency department visits found that patients who filled opioids used only between one‐quarter to one‐third of their supply [15, 16]. Previous studies have also shown that variations in opioid prescribing patterns are sometimes explained by prescriber—rather than patient—characteristics, including prescriber rank [17, 18], specialty [19] and time pressure [20]. A survey of 500 physicians about their choice of pain management in the immediate post‐operative period found that previous clinical experiences were the most commonly cited motivation for choice of medication, more so than surgery type, adherence to clinical practice guidelines or a review of relevant literature [21].

### 1.2 Importance

Currently, there is limited evidence regarding prescribing patterns for opioid‐naive populations in Canada, particularly in the province of Nova Scotia. Previous studies focused on Quebec, Alberta and Ontario [22, 23, 24]. Few studies described patterns of prescribing to populations with pain regardless of setting or pain acuity [7, 11], or for restricted populations in a single setting [25]. Estimating the prevalence of prescribing in excess of 1 week or for high‐dose or long‐acting formulations will contribute to our understanding about safety of prescribing to opioid‐naive populations in acute care settings, and potentially about their unique needs. Setting and provider specialty can serve as proxies for common clinical and training experiences among physician groups, and they can be explored as potential determinants of differences in prescribing patterns.

### 1.3 Goals of this study

The objectives of this study are to: (i) describe patterns of opioid prescribing to opioid‐naive adults who fill opioid prescriptions in community pharmacies after surgical or emergency care in Nova Scotia, Canada; (ii) determine the prevalence of filling prescriptions that are >7 days, ≥90 morphine milligram equivalent (MME)/day or for long‐acting opioids (primary outcomes), strong opioids or tramadol (secondary outcomes); and (iii) determine whether setting—for all subjects—and provider specialty—for those who had procedures—are associated with these outcomes adjusting for patient characteristics and procedure type.

## 2 METHODS

### 2.1 Study design and setting

We conducted a cross‐sectional, population‐based study in the province of Nova Scotia, Canada, using individual‐level data linked across the following five routinely collected administrative databases: the provincial Drug Information System (DIS), MSI Physicians' Billings (MSI) and Insured Patient Registry (MASTER) databases; and the national Canadian Institute for Health Information Discharge Abstract Database (CIHI‐DAD), and CIHI National Ambulatory Care Reporting System (NACRS)—(Data S1). Databases were linked using unique encoded identifiers at Health Data Nova Scotia, Dalhousie University. A de‐identified dataset was made accessible to the research team—obtaining informed consent was considered impracticable. The study was approved by the Health Sciences Research Ethics Board at Dalhousie University (REB# 2019‐4896).

### 2.2 Selection of study population

We included all adults (≥18 years) in the province of Nova Scotia, Canada, who were opioid‐naive and filled opioid prescriptions in community pharmacies between 26 April 2017 and 31 March 2019 within 14 days of having procedures or visiting and emergency departments (ED) that report to NACRS. We defined naivety as no prescription opioid fills in 180 days preceding a first fill and no fill for an opioid use disorder medication in the first three subsequent fills, if additional opioid prescriptions were filled by the subject. Using the DIS database, we identified all opioid prescriptions that were filled by adults within the study period, then excluded: (i) those who had another opioid fill in the preceding 180 days; (ii) fills for opioid formulations treating cough, diarrhoea or opioid use disorder; and (iii) fills without active insurance in the 12‐month look‐back period—opioid codes in Data S2. After identifying fills of relevance, the DIS database was linked to MSI, CIHI‐DAD and NACRS databases, and those that did not have at least one code for a surgical procedure or ED visit in the 14‐day look‐back period were excluded, similar to previous studies considering fills up to 2 weeks to be directly related to the acute pain event [26, 27] (codes in Data S3).

### 2.3 Study variables

#### 2.3.1 Characteristics of filled prescriptions and study outcomes

The primary outcomes of interest were fills for prescriptions >7 days' supply, fills for prescriptions ≥90 MME/day and fills for long‐acting opioids. Secondary outcomes were prescription fills for strong opioids and tramadol which are of concern due to potential opioid‐related harms [28] and unpredictable metabolism [29]. For each eligible subject, we obtained data from the DIS database about the first single prescription that met inclusion criteria during the study period and set the day of the fill as the subject's index day. For each fill, we obtained data about days' supply as calculated by the pharmacist and documented in the DIS database, quantity, strength and formulation, which we categorised into codeine, hydromorphone, oxycodone, morphine, tramadol or other. We also mapped opioids based on form of release (short vs. long‐acting), and potency (weak vs. strong)—see Data S4. We calculated dose in MME using the formula and conversion factors provided by the Ontario Drug Policy Research Network [30]. For subjects with multiple opioid fills on index day, MME was summed across all filled prescriptions to calculate total MME, and then divided by the number of days of the prescription with the longest days' supply. Long‐acting formulations were prioritised in the opioid type variable when both short‐ and long‐acting formulations were filled. See Data S4 for details.

#### 2.3.2 Setting of care and specialty of procedure provider (exposures)

Based on identified codes in the 14‐day look‐back period, each included subject was categorised into one of three mutually exclusive setting groups: surgical care (at least one procedure code with no ED visit codes), emergency care (at least one ED visit code with no procedure codes) or emergency plus surgical care (at least one ED visit and one procedure code). We derived a variable for specialty of procedure provider using data from the MSI database, which we categorised into general surgery, orthopaedic surgery, plastic surgery, otolaryngology, urology, other surgical specialties (cardiac‐, neuro‐, thoracic‐ and vascular‐surgery), general practice, and non‐surgical non‐general practice specialties including medical and interventional radiology specialties. Similar specialties with small frequencies were grouped, as presented.

#### 2.3.3 Patient characteristics and type of procedure (co‐variates)

We used data about patient age (continuous, in years) and sex (binary, female or male) on day of opioid filling, and binary variables (yes or no) using inpatient and outpatient ICD‐9‐CM and ICD‐10‐CA codes measuring the following conditions in the past 12‐months: depression, anxiety, alcohol, drug and tobacco abuse, low back pain, headache, arthritis, fibromyalgia, neuropathic pain and cancer, except non‐melanoma skin cancer (codes in Data S5). For subjects who had procedures, we created a procedure type variable categorised into major surgery; minor surgery; fracture, dislocation or cast; bone grafting; obstetrics; and other (included procedures that were captured in DAD but not MSI database). When a subject received multiple procedures in the 14‐day look‐back period, we used the procedure that was most proximal to fill date to create the variable. All of these factors were selected as co‐variates for adjustment in the analysis based on previous studies indicating that procedure type [21] and perception of patient behaviours and consequences [31]—which may be directly related to history of co‐morbidities included—influence physicians' prescribing decisions for pain management.

### 2.4 Statistical analysis

We summarised characteristics of the study sample and filled prescriptions overall and across settings using means with standard deviations and medians with interquartile ranges for continuous variables, and frequencies with percentages for categorical variables. We estimated the prevalence of the outcomes within each care setting and provider specialty group by estimating the proportion of individuals who had fills >7 days' supply, ≥90 MME/day or were for long‐acting opioid formulations, and assessed association of setting and provider specialty with these outcomes using multivariable logistic regression models estimating odds ratios with 95% confidence intervals (CI). Co‐variates included for adjustment are mentioned above. We considered an association to be of potential importance when the odds ratio was at least of moderate size (odds ratio ≥1.5) [32] and the confidence interval did not cross the null value. We estimated adjusted prevalences (predictive margins) at the means of covariates [33]. To enhance interpretability, adjusted prevalences were expressed as the number of patients per 1000. We conducted sensitivity analyses to test alternative definitions of acute pain (only subjects without history of cancer; only subjects who filled prescription opioids ≤2 days from emergency care), alternative categorisation of provider specialty (general practice recategorised to second listed surgical specialty), and outcomes defined as >3 days' supply, >14 days' supply and ≥50 MME/day. We performed all data management and analyses using Stata‐V15 [34].

## 3 RESULTS

### 3.1 Characteristics of the study population and filled opioid prescriptions

**FIGURE 1:**
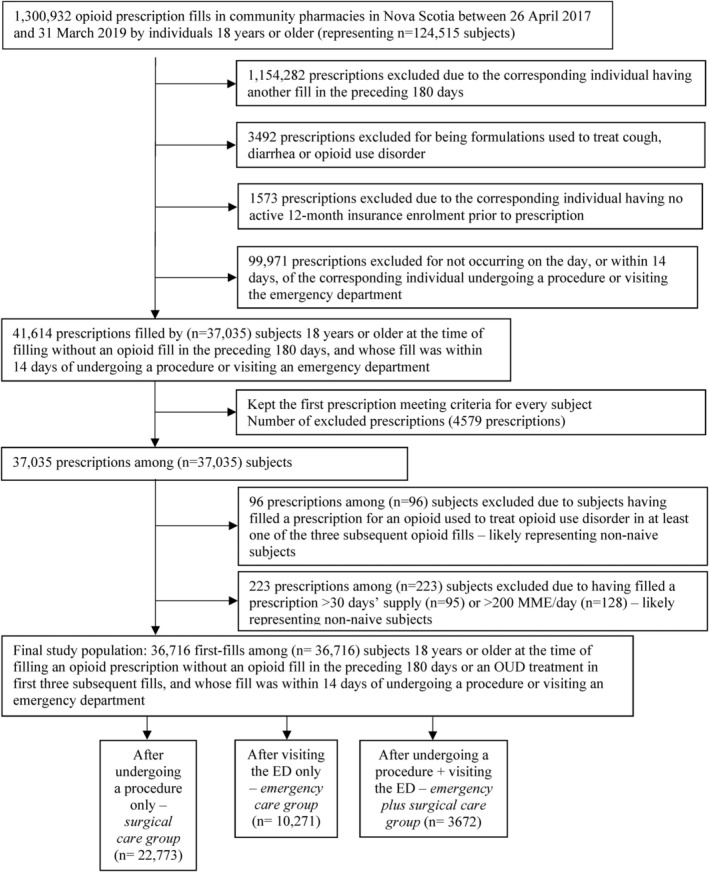
Study cohort creation flow diagram. ED, emergency department; MME, morphine milligram equivalent; OUD, opioid use disorder.

Of 124,515 adults who filled opioid prescriptions in community pharmacies in Nova Scotia between 26 April 2017 and 31 March 2019, 36,716 subjects were opioid‐naive and met the study inclusion criteria (Figure 1). Sixty‐two percent filled a prescription after surgical care, 28% after emergency care, and 10% after emergency plus surgical care. The mean age (±SD) of subjects was 54.2 ± 17.5 years and 55% were female. A past 12‐months history of arthritis, joint or neck pain diagnosis was present in 56.5%, low back pain in 17.6%, anxiety in 15.5%, depression in 10.3%, and cancer in 14.7%. Among 26,445 subjects in the surgical care and emergency plus surgical care groups 77.7% had major surgery. The most common provider billing specialties in the surgical care and emergency plus surgical care groups were general practice (34.6%), orthopaedic surgery (16.3%), general surgery (15.3%), and obstetrics and gynaecology (10.2%)—see Table 1.

**TABLE 1:** Characteristics of the study population of opioid‐naive adults who filled opioid prescriptions in community pharmacies within 14 days of surgical or emergency care in Nova Scotia, Canada (26 April 2017 to 31 March 2019) (*n* = 36,716; column percentages presented).

		Overall (*n* = 36,716 unless otherwise indicated)		Surgical care (*n* = 22,773)		Emergency care (*n* = 10,271)		Emergency plus surgical care (*n* = 3672)	
		*n*	%	*n*	%	*n*	%	*n*	%
Age and sex	Age in years, mean (SD); range	54.2 (17.5); 18–104		54.4 (16.7); 18–102		55.1 (18.7); 18–103		50.3 (18.2); 18–104	
	Female	20,147	54.8	12,871	56.5	5436	52.9	1840	50.1
Co‐morbidities in past 12‐months	Hx of depression	3794	10.3	2218	9.74	1185	11.5	391	10.7
	Hx of anxiety	5683	15.5	3324	14.6	1769	17.2	590	16.1
	Hx of alcohol abuse, or mental and behavioural disorders or physical illness due to alcohol use	620	1.70	338	1.48	179	1.74	103	2.81
	Hx of drug abuse, or mental and behavioural disorders due to drug use	317	0.86	161	0.71	104	1.01	52	1.42
	Hx of tobacco abuse, or mental and behavioural disorders due to tobacco use	3051	8.31	1915	8.41	591	5.75	545	14.8
	Hx of low back pain	6461	17.6	2967	13.0	3039	29.6	455	12.4
	Hx of headache or migraine	838	2.29	499	2.19	267	2.60	72	1.96
	Hx of arthritis, joint pain or neck pain	20,735	56.5	13,703	60.2	4929	48.0	2103	57.3
	Hx of fibromyalgia	415	1.13	215	0.94	157	1.53	43	1.17
	Hx neuropathic pain	475	1.29	293	1.29	131	1.28	51	1.39
	Any cancer, except non‐ melanoma skin cancer	5368	14.7	3916	17.2	1044	10.2	408	11.1
Type of procedure, if applicable (n = 26,445)	Major surgery	20,543	77.7	18,472	81.1	–	–	2071	56.4
	Minor surgery	1207	4.56	1010	4.44	–	–	197	5.36
	Fracture, dislocation, or cast	1809	6.84	805	3.53	–	–	1004	27.3
	Bone grafting	1108	4.19	1002	4.40	–	–	106	2.89
	Obstetrics	718	2.72	710	3.12	–	–	8	0.22
	Other	1060	4.01	774	3.40	–	–	286	7.79
Procedure location, if applicable (n = 26,445)	Hospital	24,230	91.6	21,001	92.2	–	–	3229	87.9
	Office	1155	4.40	998	4.38	–	–	157	4.28
	Unknown	1060	4.00	774	3.40	–	–	786	7.79
Billing specialty of procedure provider, if applicable (*n* = 26,445)	General practice	9156	34.6	8188	36.0	–	–	968	26.4
	Orthopaedic surgery	4302	16.3	3533	15.5	–	–	769	20.9
	General surgery	4041	15.3	3181	14.0	–	–	860	23.4
	Obstetrics and gynaecology	2709	10.2	2566	11.3	–	–	143	3.89
	Plastic surgery	1508	5.70	1269	5.57	–	–	239	6.51
	Otolaryngology	1264	4.78	1181	5.19	–	–	83	2.26
	Urology	1208	4.57	1041	4.57	–	–	167	4.55
	Other surgical specialties	898	3.40	788	3.46	–	–	110	3.00
	Other non‐surgical specialties	299	1.13	252	1.11	–	–	47	1.28
	Unknown	1060	4.01	774	3.40	–	–	286	7.79

*Note*: Column percentages presented. Only ‘yes’ category presented for binary variables; all categories presented for variables with 3+ categories. Undergoing procedures identified from MSI Physicians' Billings using Canadian Classification of Procedures (CCP) codes and Canadian Institute for Health Information Discharge Abstract Database (CIHI‐DAD) using the Canadian Classification of Health Interventions codes after linkage to Drug Information System Database (DIS). Emergency department visits identified from the National Ambulatory Care Reporting System database after linkage to DIS. Age in years on day of index opioid filling. 12‐month history of mental health illness, substance use, chronic pain and cancer identified using International Classification Diseases 9th edition (ICD‐9‐CM) diagnostic codes and International Classification Diseases 10th edition Canadian enhanced version (ICD‐10‐CA) diagnostic codes in MSI Physicians' Billings and CIHI DAD databases, respectively. See Data S5 for full list of ICD codes. Type of procedure identified using CCP codes in MSI Physicians' Billings Database; billing specialty of procedure provider identified from MSI Physicians' Billings Database.

Abbreviations: Hx, 12‐month past history; m, months.

Overall, hydromorphone was the most filled opioid (50%; 18,370 subjects), followed by codeine (26.4%), tramadol (8.5%), morphine (7.8%) and oxycodone (7.0%). Median days' supply and dose were 3 days (IQR 2–5) and 50 MME/day (IQR 30–75). Less than 1% (251 subjects; 0.7%) filled multiple prescriptions on index day—see Table 2 for characteristics across settings.

**TABLE 2:** Characteristics of first‐prescription fills in community pharmacies in Nova Scotia (26 April 2017 to 31 March 2019) by opioid‐naive adults who filled opioid prescriptions within 14 days of surgical or emergency care in Nova Scotia, Canada, and prevalence of outcomes (*n* = 36,716; column percentages presented).

				Overall (*n* = 36,716)		Surgical care (*n* = 22,773)		Emergency care (*n* = 10,271)		Emergency plus surgical care (*n* = 3672)	
				*n*	%	*n*	%	*n*	%	*n*	%
Characteristics		Type of opioid	Codeine	9682	26.4	5482	24.1	3595	35.0	605	16.5
			Hydromorphone	18,370	50.0	12,706	55.8	3215	31.3	2449	66.7
			Oxycodone	2579	7.02	1799	7.90	562	5.47	218	5.94
			Morphine	2876	7.83	1130	4.96	1503	14.6	243	6.62
			Tramadol	3113	8.48	1605	7.05	1355	13.2	153	4.17
			Other	96	0.26	51	0.22	41	0.40	<5^a^	0.11
		Route	Oral	36,444	99.3	22,715	99.8	10,077	98.1	3652	99.5
			Other	272	0.74	58	0.20	194	1.89	20	0.50
		Days' supply	Median; range (IQR)	3; 1–30	(2–5)	3; 1–30	(2–5)	4; 1–30	(2–5)	3; 1–30	(2–4)
			≤3 days	20,650	56.2	13,106	57.6	5131	50.0	2413	65.7
			4–7 days	12,082	32.9	7678	33.7	3401	33.1	1003	27.3
			>7 days	3984	10.9	1989	8.73	1739	16.9	256	6.97
		MME	Total, median (IQR)	150	(100–300)	150	(100–300)	135	(90–200)	150	(100–300)
			Daily, median (IQR)	50	(30–75)	50	(37.5–83.3)	36	(22.5–50)	50	(36–100)
		Multiple fills on index day	Yes	251	0.68	123	0.54	96	0.93	32	0.87
		ED visit to fill, if applicable	Days, median (IQR)	–	–	–	–	1	(0–2)	3	(1–6)
Outcomes	Main	>7 days' supply	Yes	3984	10.9	1989	8.73	1739	16.9	256	6.97
		≥90 MME/day	Yes	7369	20.2	5506	24.2	909	9.02	954	26.1
		Long‐acting	Yes	266	0.72	151	0.66	104	1.01	11	0.30
	Secondary	Strong opioid	Yes	23,921	65.2	15,686	68.9	5321	51.8	2914	79.4
		Tramadol	Yes	3113	8.48	1605	7.05	1355	13.2	153	4.17
	Alternative	>3 days' supply	Yes	16,066	43.8	9667	42.5	5140	50.0	1259	34.3
		>14 days' supply	Yes	1257	3.42	454	1.99	722	7.03	81	2.21
		≥50 MME/day	Yes	18,930	51.9	13,511	59.5	3141	31.2	2278	62.4

*Note*: Strong opioids: Hydromorphone, oxycodone, morphine, fentanyl; Weak opioids: Codeine, tramadol. MME total and per day are calculated for oral opioid formulations (*n* = 36,444; 99.3% of cohort). This is the n included in all dose variables.

Abbreviations: ED, emergency department; IQR, interquartile range; MME, morphine milligram equivalents.

^a^ Cells with *n* < 5 are indicated as such in the table for privacy.

### 3.2 Prevalence of outcomes and association with setting and specialty of procedure provider

**FIGURE 2:**
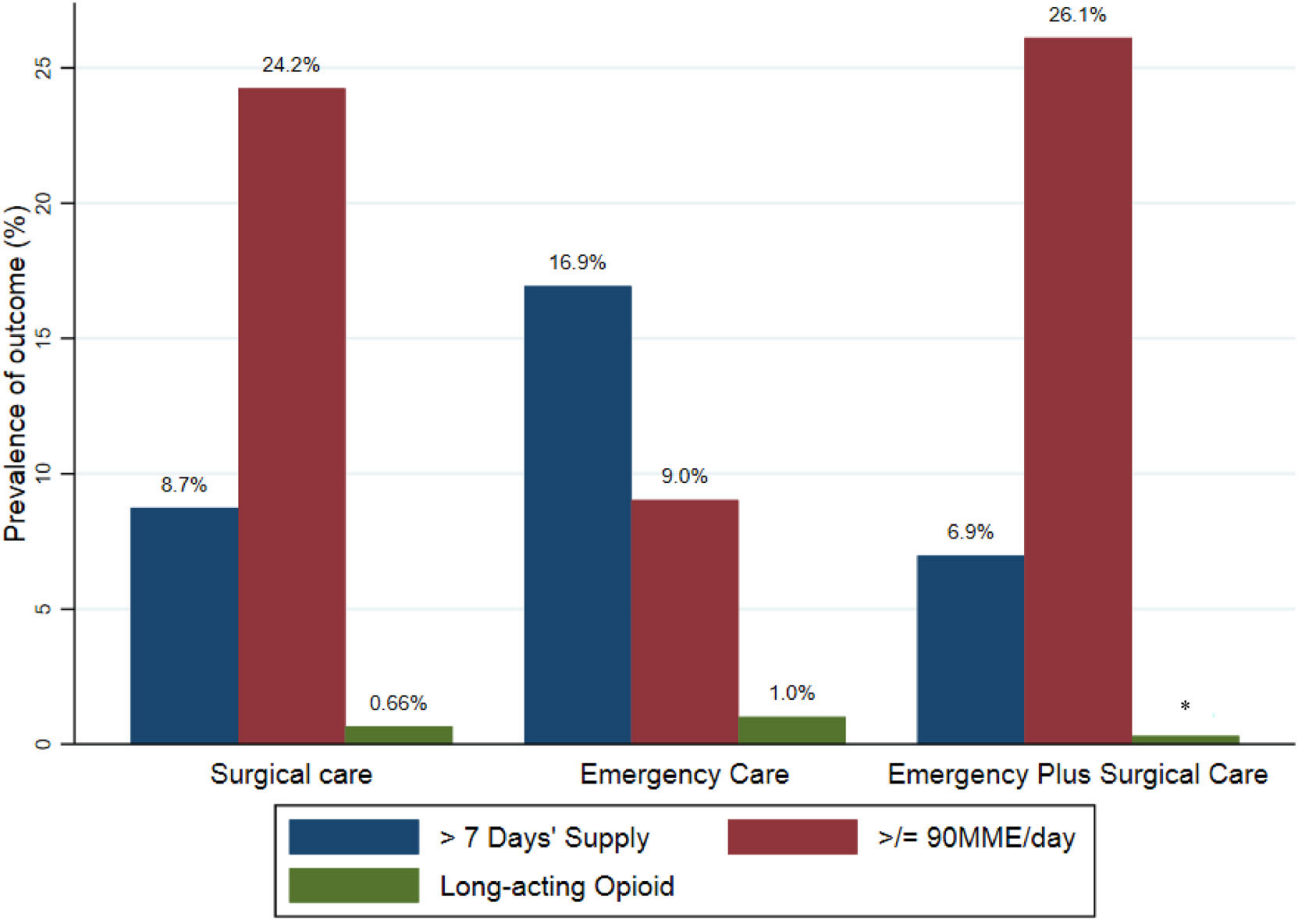
Prevalence of filling prescriptions >7 days' supply, ≥90 MME/day and for long‐acting opioids across care settings. (*n* = 36,716). MME, morphine milligram equivalent. *Suppressed value (*n* < 5).

Overall, prescriptions >7 days' supply were filled by 10.9% (*n* = 3984), ≥90 MME/day by 20.2% (*n* = 7369) and 0.7% filled prescriptions for long‐acting opioids (*n* = 266)—see Table 2 and Figure 2 for prevalence across settings. Analyses adjusting for patients' age, sex and 12‐month history of depression, anxiety, alcohol abuse, drug abuse, tobacco abuse, low back pain, headache, arthritis, fibromyalgia, neuropathic pain, and cancer, showed that compared to the surgical care group, the emergency care group was 2 times as likely have filled prescriptions >7 days' supply (adjusted odds ratio [aOR] 2.13, 95% 1.98–2.29; absolute adjusted risk 87.1/1000 in surgical care and 166/1000 in emergency care) but less likely have filled ≥90 MME/day (aOR 0.36, 95% CI 0.34–0.39—absolute adjusted risk 237/1000 in surgical care and 97/1000 in emergency care). Those in the emergency plus surgical care group were less likely to have filled prescriptions >7 days' supply (aOR 0.86, 95% CI 0.75–0.99; absolute adjusted risk 76/1000) and slightly more likely to have filled prescriptions ≥90 MME/day (aOR 1.07, 95% CI 0.99–1.16; absolute adjusted risk 249/1000) compared to the surgical group—Table 3.

**TABLE 3:** Unadjusted and adjusted odds ratios (with 95% CI) for filling prescriptions >7 days' supply, ≥90 MME/day, or for long‐acting opioids based on setting and specialty of procedure provider among opioid‐naive patients who had surgical or emergency care and filled opioid prescriptions between 26 April 2017 and 31 March 2019 in community pharmacies in Nova Scotia, Canada (setting *n* = 36,716; billing specialty *n* = 26,445; row percentages presented).

		>7 days' supply = yes (*n* setting = 36,716; *n* specialty = 26,445)			≥90 MME = yes (*n* setting = 36,716; *n* specialty = 26,445)			Long‐acting opioid = yes (*n* setting = 36,716; *n* specialty = 26,445)		
		*n* (row %)	OR (95% CI)	aOR (95% CI)	*n* (row %)	OR (95% CI)	aOR (95% CI)	*n* ^a^ (row %)	OR (95% CI)	aOR (95% CI)
Setting (*n* = 36,716)	Surgical care (*n* = 22,773)	1989 (8.73)	Ref	Ref	5506 (24.2)	Ref	Ref	151 (0.66)	Ref	Ref
	Emergency care (*n* = 10,271)	1739 (16.9)	2.13 (1.99–2.28)	2.13 (1.98–2.29)	909 (9.02)	0.31 (0.29–0.33)	0.34 (0.31–0.37)	104 (1.01)	1.53 (1.19–1.97)	1.36 (1.04–1.77)
	Emergency plus surgical care (*n* = 3672)	256 (6.97)	0.78 (0.68–0.90)	0.86 (0.75–0.99)	954 (26.1)	1.11 (1.02–1.20)	1.07 (0.99–1.16)	<5^a^	0.45 (0.24‐0.83)	0.44 (0.23–0.82)
Billing specialty of procedure provider, for all subjects who had procedures (*n* = 26,445)	General surgery (*n* = 4041)	141 (3.51)	Ref	Ref	501 (12.4)	Ref	Ref	43 (1.06)	Ref	Ref
	Orthopaedic surgery (*n* = 4302)	284 (6.60)	1.94 (1.57–2.39)	1.17 (0.94–1.47)	1675 (39.0)	4.52 (4.04–5.05)	2.92 (2.58–3.30)	5 (0.12)	0.11 (0.04–0.27)	0.17 (0.06–0.45)
	Obstetrics and gynaecology (*n* = 2709)	125 (4.61)	1.33 (1.04–1.70)	1.46 (1.13–1.89)	526 (19.5)	1.71 (1.49–1.95)	1.94 (1.68–2.22)	0 (0.00)	‐	‐
	Plastic surgery (*n* = 1508)	64 (4.24)	1.22 (0.90–1.64)	1.03 (0.75–1.40)	182 (12.1)	0.97 (0.81–1.16)	0.74 (0.62–0.89)	<5^a^	0.12 (0.03–0.51)	0.16 (0.04–0.67)
	Otolaryngology (*n* = 1264)	187 (14.8)	4.77 (3.79–5.99)	4.89 (3.86–6.20)	95 (7.52)	0.57 (0.46–0.72)	0.63 (0.50–0.79)	<5^a^	0.07 (0.01–0.54)	0.07 (0.01–0.50)
	Urology (*n* = 1208)	54 (4.47)	1.28 (0.93–1.77)	1.19 (0.86–1.65)	109 (9.08)	0.70 (0.57–0.88)	0.68 (0.54–0.85)	13 (1.08)	1.01 (0.54–1.89)	0.90 (0.48–1.70)
	Other surgical specialties (*n* = 898)	85 (9.47)	2.87 (2.17–3.79)	2.52 (1.89–3.36)	202 (22.5)	2.05 (1.71–2.47)	1.52 (1.26–1.84)	<5^a^	0.10 (0.01–0.75)	0.09 (0.01–0.70)
	General practice (*n* = 9156)	1059 (11.6)	3.59 (3.00–4.30)	2.31 (1.91–2.79)	2798 (30.6)	3.11 (2.81–3.45)	2.45 (2.20–2.74)	65 (0.71)	0.66 (0.45–0.98)	0.89 (0.59–1.36)
	Non‐surgical non general practice specialties (*n* = 299)	92 (30.8)	12.2 (9.06–16.4)	6.66 (4.87–9.11)	25 (8.53)	0.66 (0.43–1.00)	0.74 (0.48–1.13)	9 (3.01)	2.89 (1.39–5.98)	2.12 (0.98–4.60)
	Unknown^b^ (*n* = 1060)	153 (14.4)	4.63 (3.65–5.88)	5.35 (4.19–6.85)	347 (33.3)	3.53 (3.01–4.14)	3.06 (2.60–3.60)	23 (2.17)	2.06 (1.24–3.44)	2.10 (1.24–3.55)

*Note*: Row percentages presented. Undergoing procedures identified from MSI Physicians' Billings using Canadian Classification of Procedures codes and Canadian Institute for Health Information Discharge Abstract Database using the Canadian Classification of Health Interventions (CCI) codes after linkage to Drug Information System Database (DIS). Emergency department visits identified from National Ambulatory Care Reporting System database after linkage to DIS. Billing specialty of procedure provider identified from MSI Physicians' Billings Database. Other surgical specialties are cardiac surgery (*n* = 135), neurosurgery (*n* = 229), thoracic surgery (*n* = 389) and vascular surgery (*n* = 151). Non‐surgical non general practice specialties ranged from *n* = 1 to *n* = 144 in the following specialties: anaesthesia, cardiology, dermatology, diagnostic radiology, emergency medicine, gastroenterology, haematology, internal medicine, medical oncology, ophthalmology, optometry, pathology, psychiatry, radiation oncology and respiratory medicine. Covariates included in the adjusted models: patient age (continuous, in years) and sex (binary, female or male) on the day of opioid filling, binary variables (yes vs. no) measuring 12‐month history of the following conditions: depression, anxiety, alcohol abuse, drug abuse, tobacco abuse, low back pain, headache, arthritis, fibromyalgia, neuropathic pain and cancer (any cancer except non‐melanoma skin cancer)—codes in Data S5. In the model for specialty of procedure provider, we also adjusted for procedure type (major surgery; minor surgery; fracture, dislocation, or cast; bone grafting; obstetrics; and other, which included procedures that were captured in Discharge Abstract Database database but not MSI).

Abbreviations: aOR, adjusted odds ratio; CI, confidence interval; MME, morphine milligram equivalent; OR, odds ratio.

^a^ Cells with *n* < 5 are indicated as such in the table for privacy.

^b^ Billing specialty of procedure provider not available (CCI code).

**FIGURE 3:**
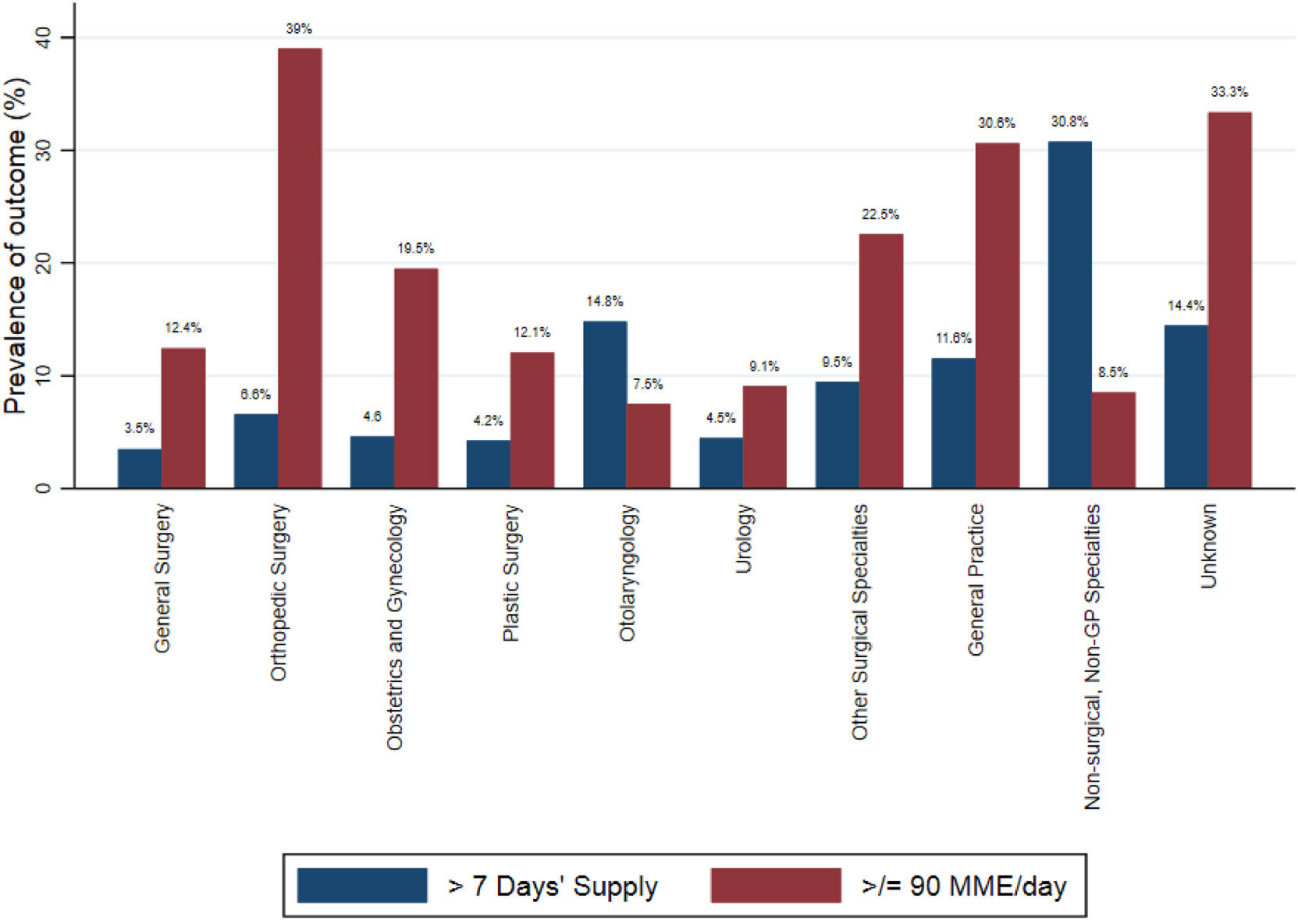
Prevalence of filling prescriptions >7 days' supply and ≥ 90 MME/day across provider specialty groups for subjects who had procedures (surgical and emergency plus surgical care groups). (*n* = 26,445). MME, morphine milligram equivalent.

Prevalence of filling prescriptions >7 days' supply and ≥ 90 MME/day across specialty groups is presented in Table 3 and Figure 3. Among subjects in the surgical care and emergency plus surgical care groups (*n* = 26,445), 15.2% (*n* = 4041) had general surgeons for providers. Using this group as reference and after adjusting for patient characteristics and procedure type, subjects in the following specialties had potentially important higher odds of filling prescriptions >7 days' supply: otolaryngology (aOR 4.77, 95% CI 3.79–5.99), other surgical specialties including cardiac‐, neuro‐, thoracic‐ and vascular‐surgery (aOR 2.52, 95% CI 1.89–3.36), general practice (aOR 2.31 95% CI 1.91–2.79), and non‐surgical non‐general practice specialties (aOR 6.66, 95% CI 4.87–9.11). Subjects in the following specialties also had potentially important higher odds of filling prescriptions ≥90 MME/day compared to general surgery: orthopaedic surgery, obstetrics and gynaecology, other surgical specialties, and general practice, while the following specialties had lower odds: plastic surgery, otolaryngology and urology (Table 3).

### 3.3 Prevalence of filling strong opioids and tramadol (secondary outcomes)

Just below two‐thirds of the study population filled prescriptions for strong opioids (65.2%) and 8.5% for tramadol (Table 2). Adjusted analyses showed that the emergency care group had a potentially important higher odds of filling tramadol compared to surgical care (aOR 1.79, 95% CI 1.66–1.94) and other potentially important differences across settings and procedure provider specialty groups were also observed (results in Data S6).

### 3.4 Sensitivity analyses

The analysis that included only subjects without history of cancer in the past 12 months showed similar results. Analysis that included only subjects who filled their prescription at ≤2 days from emergency care showed a weaker association between emergency care and >7 days' supply outcome. Recategorising those whose provider specialty was general practice to second listed surgical specialty showed stronger associations, particularly with >7 days' supply and long‐acting opioids outcomes. Redefining outcomes as >3 days' supply showed weaker associations, and >14 days' supply showed weaker association for otolaryngology, and stronger associations for other surgical, and non‐surgical non‐general practice specialties (results in Data S7).

## 4 DISCUSSION

We found that prescriptions that were filled by previously opioid‐naive subjects after surgical and emergency care in Nova Scotia were written, on average, for 3 days (IQR 2–5) and 50 MME/day (IQR 30–75), and that half of this population filled hydromorphone formulations, while a quarter filled codeine formulations. We also found that one in 10 of the study population had prescriptions that exceeded 7 days' supply, around one in five exceeded 90 MME/day, while on <1% filled long‐acting formulations. We also found that, after accounting for measured patient characteristics, distinct patterns of prescribing were observed. Subjects who received opioids after emergency care were twice as likely to have long days' supply prescriptions but less than half as likely to fill ≥90 MME/day compared to those who received surgical care. Furthermore, we found that among those who filled opioids after surgical care, large relative differences in filling long days' supply and high‐dose prescriptions existed across provider specialty groups. Those who filled prescriptions after a procedure that was billed by otolaryngology had the highest likelihood of filling prescriptions longer than 1 week's supply among the surgical subspecialties, while prescriptions ≥90 MME/day were most likely to be filled after a procedure that was billed by orthopaedic surgery.

Overall, our study findings on differences in prescribing for patients receiving surgical care across provider specialty groups independent of patient characteristics aligns with recent studies of opioid‐naive surgical patients from the United States which found variation in opioid prescribing by provider rank and specialty independent of patient factors [17, 18, 19].

The tendency for prolonged opioid prescriptions (>7 days) in otolaryngological, cardiac, neuro, thoracic and vascular surgeries may be influenced by the complex nature of these procedures and associated pain levels, extended recovery times, and the unique prescribing cultures within these fields. Surgeons in these specialties may be more likely to prescribe longer prescriptions, reflecting a clinical practice aimed at avoiding under‐treatment of pain, which, in turn, contributes to potential overprescription. Differences between these specialties and general surgery in duration to post‐op follow‐up visit, where the expected return to see the surgeon after surgery may be longer, particularly after minor procedures which frequently occur in otolaryngology could also contribute to the observed findings. This may also be the case in the emergency setting where long days' supply is observed. In emergency settings, longer prescriptions might reflect an attempt to prevent patients from returning for insufficient pain relief, particularly in the context of a lack of access to primary care physicians for follow‐up. Previous studies found that physicians frequently overprescribe opioids in emergency and surgical settings [15, 16] in an attempt to ensure patients have sufficient supply ‘just in case’.

By contrast, specialties such as orthopaedics, which were more likely to be associated with higher opioid dosages (>90 MME/day), may have distinct prescribing patterns that emphasise managing intense, short‐term postoperative pain with higher doses, reflecting a different approach compared to the extended‐duration prescribing patterns seen in more complex surgical specialties. This distinction points to the potential impact of prescribing cultures and established clinical protocols within each specialty. Obstetrics and gynaecology, cardiac, neuro, thoracic and vascular surgeries were linked to both prolonged opioid use (>7 days) and high doses (>90 MME/day), potentially due to a combination of high‐intensity pain, complex recovery and a prescribing culture that prioritises ensuring adequate pain management in these patients. These specialties may have a higher threshold for pain control, leading to practices that contribute to prolonged and higher‐dose opioid prescriptions.

Another explanation for the observed differences could be variation in average length of stay at the hospital following procedures, and subsequently differences in amount of inpatient opioid prescribing that we could not measure or account for in the study. Still, unmeasured confounding by patients' clinical need could explain the observed variation, and this needs to be explored in future studies.

All of the above proposed considerations, highlight the complexity of prescribing decisions and underscore the importance of understanding healthcare context and patient follow‐up pathways. It further underscores the need for further exploration of the motives behind physicians' prescribing decisions. Furthermore, these findings indicate that developing opioid‐prescribing guidelines that are tailored to the specific needs of each setting and surgical subspecialty, rather than relying on broad, generalised guidelines, may be needed to address the varying pain management and practical care delivery requirements across different specialties adequately.

The differences in opioid prescription patterns between surgical and emergency care, with observed considerable variation within the surgical setting itself, indicate that medical bodies should encourage practices of routine auditing of opioid prescribing and explore whether variations in prescribing practices align with medical necessity. Norms in routine postoperative opioid prescribing should be interrogated at medical specialty and subspecialty level to ensure that genuine patient need, and not routine practice norms, are dictating prescribing patterns. Overall, policies should encourage that when considering pain management for surgery‐related conditions, clinicians should balance between sufficient pain relief and opioid stewardship. From a public health policy perspective, these findings could inform the development of more tailored opioid prescribing guidelines that cater to specific surgical specialties and their prevalent pain aetiologies, aiming to reduce the risk of long‐term opioid use.

Beyond the recommendations published in 2017 [35], no new Canadian opioid prescribing guidelines have been introduced since the period of our study (2017–2019). The recent 2022 CDC guidelines in the United States [36] continue to emphasise careful opioid prescribing practices similar to those that were already in place during our study period, suggesting that our findings remain relevant in contemporary clinical contexts. In Nova Scotia, a provincial Prescription Monitoring Program and physician mentorship and training programs for general practitioners are in place to guide safer opioid prescribing [37]. While our study period predated the expansion of some of these efforts, these programs may currently influence prescribing patterns by providing feedback to prescribers, identifying over‐prescribing behaviour and potentially reducing inappropriate opioid use. Future evaluations of these programs could assess their effects on prescribing practices and determine whether they mitigate some of the patterns observed in our study.

Judicious prescribing of opioids for the treatment of acute pain was identified as a priority to combatting the opioid epidemic [38, 39]. Recent evidence shows that institutional regulation and policy legislation can effectively drive down prescribing when appropriate [40, 41]. Before determining targets for intervention in care settings and specialties, it is essential to explore possible explanations for variation as previously described. Some patients may benefit from higher dose or long days' supply prescriptions, such as those with severe pain requiring adequate analgesia for early mobilisation or those with long expected recovery duration. Therefore, it is important to gain more insight for this population before making recommendations to change practice to avoid causing undue restriction on prescribing.

Our study has some limitations that must be noted. The definition of opioid‐naivety may have missed patients who use illicitly manufactured opioids or acquire prescription opioids through diversion. A study in the United States found that six out of 82 surgical patients who did not use prescription opioids in the past reported using illicitly manufactured opioids [42]. We identified a small number of subjects who filled formulations used to treat opioid use disorder in their first three subsequent fills, considered them likely non‐naive, and excluded them. The indicator for ED visits captured only 52% of ED visits in the province of Nova Scotia in 2017–2018 [2]. This may have excluded half of eligible subjects in this setting and the prevalence of outcomes for them remains to be estimated. The data we used did not take into account the length of hospital stay after surgery. Those who had an opioid prescribed and filled after surgery, but had a long hospital stay (>14 days), would not have been included in the current study. However, we expect that the proportion of those individuals is minimal as the average length of stay post‐operatively in Canada for the 10 most common surgeries ranges between 2.1 and 10.6 days [43]. To assess the study outcomes, we used data from drug claims which do not include information on inpatient prescriptions or prescriptions that were given but never filled. If those who do fill their prescriptions are more likely to have had high‐dose and long days' supply prescriptions compared to those who do not fill them, then we might observe an over‐estimation of the prevalence of these outcomes in the study. We did not include a comorbidity index as a covariate in our multivariable models, which could have further mitigated residual confounding. Although multimorbidity indices may outperform individual comorbidities in prognostic models [44], their advantages over individual comorbidities in exploratory cross‐sectional analyses like ours are less established. However, by adjusting for 11 specific comorbidities, our models minimised confounding in the association analyses as much as possible. Similarly, while our models were adjusted for patients' demographics, historical conditions and procedure types, we did not include discharge medications as covariates due to limited available data. Including this information in future analyses could provide a more comprehensive understanding of the factors contributing to postoperative opioid use. Co‐morbidity measures may have misclassified subjects if they were not yet diagnosed or if they were using prescription medications but did not have corresponding ICD codes documented in included databases during the 12‐month look‐back period. These situations, in addition to potentially having missed other important confounders, leaves the possibility of residual confounding in the study that may partially or fully explain the differences in prescribing observed here. Finally, we cannot completely rule out the possibility that the 2017 Canadian opioid guidelines influenced prescribing practices [35]. However, most participants in our study were from 2018 and 2019, providing more contemporary estimates. While temporal trends may exist, their impact is likely to be reasonably uniform across all settings, suggesting that any potential temporal biases have likely been minimised in our comparisons between different settings and specialties.

In summary, this study found that overall, the majority of opioid prescriptions filled by opioid‐naive patients following surgical and emergency care in Nova Scotia were below 7 days' supply and 90 MME/day. One in 10 had a prescription that exceeded 7 days' supply, and one in five had a prescription ≥90 MME/day. We found variation across settings and provider specialty groups in these outcomes. We recommend exploring drivers of the observed variation and whether these patterns of prescribing are explained by differences in patients' clinical needs or if other factors, such as prescribing cultures, account for these differences.

## AUTHOR CONTRIBUTIONS

Roah A. Merdad conceived the study with advice from Mark Asbridge, Samuel Campbell and Jill A. Hayden; Roah A. Merdad and Jill A. Hayden designed the study with input from Mark Asbridge; Roah A. Merdad managed and analysed data with input from Jill A. Hayden, Mark Asbridge and Daniel J. Dutton; Roah A. Merdad drafted the manuscript; Mark Asbridge, Samuel Campbell, Daniel J. Dutton and Jill A. Hayden all contributed significantly to its revision. Roah A. Merdad takes responsibility for the manuscript as a whole.

## FUNDING INFORMATION

A PhD thesis bursary provided by the Saudi Arabian Cultural Bureau fully covered data access expenses for this research.

## CONFLICT OF INTEREST STATEMENT

The authors have no conflicts of interest to declare.

## ETHICS STATEMENT

This study was approved by the Health Sciences Research Ethics Board at Dalhousie University (REB# 2019–4896).

## Supporting information


**DATA S1.** Supporting information.
