# Supplementary material for: Patterns of opioid prescribing to opioid‐naive patients after surgical and emergency care: A population‐based cross‐sectional study using linked administrative databases in Nova Scotia (2017–2019)
Source: Drug Alcohol Rev. 2025 Mar 7;44(4):1124–37. doi: 10.1111/dar.14029 (PMC12117293; doi:10.1111/dar.14029)
Supplement: Supplementary file 1 — DATA S1. Supporting information. [file DAR-44-1124-s001.docx]

**Patterns of opioid prescribing to opioid naive patients after surgical and emergency care: a population-based cross-sectional study using linked administrative databases in Nova Scotia (2017 - 2019).**

**SUPPLEMENTARY MATERIAL**

*Running Title: Opioid prescribing to opioid naive patients after surgical and emergency*

**Appendix 1. Data sources**

**Appendix 2. List of opioid DINs**

**Appendix 3. List of procedure and emergency department visit codes**

**Appendix 4. Dose calculation and opioid variable definitions**

**Appendix 5. List of ICD-9-CM and ICD-10-CA codes**

**Appendix 6. Results of secondary outcome analyses**

**Appendix 7. Results of sensitivity analyses**

**Appendix 1: Data sources**

|  | Database | Description | Population included in database | Used to identify cohort | Used to obtain data for study variables |
| --- | --- | --- | --- | --- | --- |
| 1 | **Drug Information System (DIS)** | The DIS records information on all prescriptions filled in a community pharmacy in Nova Scotia. DIS data are collected by Nova Scotia Department of Health and Wellness and is a part of Nova Scotia’s electronic health record (EHR) system. The DIS is made available to researchers in Nova Scotia through Health Data Nova Scotia (HDNS). | All individuals, excluding military, filling prescriptions from physicians in Nova Scotian community pharmacies. | √  Identify opioid fills, determine naivety status, identify formulations for exclusion | √  Date of index fill, all prescription data |
| 2 | **MSI Physician’s Billings (MSI)** | This dataset is created and maintained by Medavie Blue Cross for the Department of Health and Wellness in Nova Scotia. It contains records for each insured service encounter rendered by a physician and paid for by the Nova Scotia Medical Services Insurance (MSI). This dataset is originally gathered and used for administrative and auditing purposes, but made available to researchers in Nova Scotia through HDNS. | All individuals, excluding military, receiving billable services from physicians in Nova Scotia. | √  Identify codes for procedures to determine eligibility | √  Data on patient chronic pain, mental illness, substance abuse, and cancer diagnoses + type of procedure, billing specialty of procedure provider. |
| 3 | **CIHI Discharge Abstract Database (DAD)** | DAD captures administrative, clinical, and demographic information on hospital discharges (including deaths, sign-outs and transfers). DAD data are collected by the Canadian Institute for Health Informatics (CIHI). CIHI collects data and makes it available to stakeholders to support decision making in health care, health system performance, and population health. In Nova Scotia, it is made available to researchers through HDNS. | All individuals, excluding military, discharged from acute care facilities which submit data to the DAD | √  Identify codes for procedures to determine eligibility | √  Data on patient chronic pain, mental illness, substance abuse, and cancer diagnoses |
| 4 | **CIHI National Ambulatory Care Reporting Systems (NACRS)** | NACRS contains data for all hospital-based and community-based ambulatory care: day surgery, outpatient and community-based clinics, and emergency departments. CIHI collects data and makes it available to stakeholders to support decision making in health care, health system performance, and population health. It is made available to researchers in Nova Scotia through HDNS. | All individuals, excluding military, receiving ambulatory/emergency care, as well as day surgeries | √  Identify codes for ED visit | √  Date of visit to calculate time from visit to fill |
| 5 | **Insured Patient Registry (MASTER)** | MASTER contains demographic, geographic, and insurance (eligibility start/end dates, termination status and reason) information on the entire population of insured healthcare beneficiaries in Nova Scotia. It is used for administrative purposes by the Department of Health and Wellness and made available to researchers in Nova Scotia through HDNS. | Entire population of insured health care beneficiaries in Nova Scotia. | √  Determine whether 18+ years on day of fill | √  Age and sex variables |

**Appendix 2: Opioid DINs** used to select study cohort and measure filled index prescription.

*(Courtesy of BACK Program and Hayden et al. 2021:* Hayden JA, Ellis J, Asbridge M, Ogilvie R, Merdad R, Grant DAG, et al. Prolonged opioid use among opioid-naive individuals after prescription for nonspecific low back pain in the emergency department. Pain. 2021;162(3):740-8.*)*

| **Morphine** | | | |
| --- | --- | --- | --- |
| Morphine LP Epidural 0.5mg/mL Inj | 02021056 | M-Eslon ER 15mg Cap | 02177749 |
| Morphine LP Epidural 1mg/mL Inj | 02021048 | Kadian 20mg Cap | 02184435 |
| Morphine Sulfate 1mg/mL Inj | 01980696 | MS-IR 20mg Tab | 02014238 |
| Doloral 1mg/mL Syr | 00614491 | Statex 20mg Supp | 00596965 |
| Statex 1mg/mL Syr | 00591467 | Statex 20mg/mL Drops | 00621935 |
| Morphine Sulfate 2mg/mL Inj | 02242484 | Statex 25mg Tab | 00594636 |
| Morphine Sulfate 2mg/mL Inj | 01964437 | Morphine SR 30mg Tab | 02350890 |
| Morphine Sulfate 5mg/mL Inj | 01964429 | Novo-Morphine SR 30mg Tab | 02302772 |
| MS-IR 5mg Tab | 02014203 | Sandoz Morphine SR 30mg Tab | 02244791 |
| Statex 5mg Tab | 00594652 | MS Contin 30mg Tab | 02014297 |
| Statex 5mg/mL Syr | 00591475 | M-Eslon ER 30mg Cap | 02019949 |
| Morphine Sulfate 10mg/mL Inj | 00392588 | MS-IR 30mg Tab | 02014254 |
| Kadian 10mg Cap | 02242163 | Statex 30mg Supp | 00639389 |
| M-Eslon ER 10mg Cap | 02019930 | Morphine HP 50mg/mL Inj | 00617288 |
| MS-IR 10mg Tab | 02014211 | Kadian 50mg Cap | 02184443 |
| Statex 10mg Supp | 00632201 | Statex 50mg Tab | 00675962 |
| Statex 10mg Tab | 00594644 | Statex 50mg/mL Drops | 00705799 |
| Morphine SR 15mg Tab | 02350815 | Morphine SR 60mg Tab | 02350912 |
| Novo-Morphine SR 15mg Tab | 02302764 | Novo-Morphine SR 60mg Tab | 02302780 |
| Sandoz Morphine SR 15mg Tab | 02244790 | Sandoz Morphine SR 60mg Tab | 02244792 |
| MS Contin 15mg Tab | 02015439 | MS Contin 60mg Tab | 02014300 |
| Morphine Sulfate 15mg/mL Inj | 00392561 | M-Eslon ER 60mg Cap | 02019957 |
| Novo-Morphine SR 100mg Tab | 02302799 | Novo-Morphine SR 200mg Tab | 02302802 |
| MS Contin 100mg Tab | 02014319 | MS Contin 200mg Tab | 02014327 |
| Kadian 100mg Cap | 02184451 | M-Eslon ER 200mg Cap | 02177757 |
| M-Eslon ER 100mg Cap | 02019965 |  |  |
| **Hydromorphone** | | | |
| pms-Hydromorphone 1mg/mL Oral Sol | 01916386 | Apo-Hydromorphone 8mg Tab | 02364158 |
| Dilaudid 1mg/mL Oral Sol | 00786535 | pms-Hydromorphone 8mg Tab | 00885428 |
| Apo-Hydromorphone 1mg Tab | 02364115 | Teva-Hydromorphone 8mg Tab | 02319446 |
| pms-Hydromorphone 1mg Tab | 00885444 | Dilaudid 8mg Tab | 00786543 |
| Teva-Hydromorphone 1mg Tab | 02319403 | Jurnista 8mg Tab | 02337274 |
| Dilaudid 1mg Tab | 00705438 | Hydromorph Contin 9mg Cap | 02359510 |
| Hydromorphone 2mg/mL Inj | 02145901 | Hydromorphone HP 10mg/mL Inj | 02145928 |
| Dilaudid 2mg/mL Inj | 00627100 | Dilaudid HP 10mg/mL Inj | 00622133 |
| Apo-Hydromorphone 2mg Tab | 02364123 | Hydromorph Contin 12mg Cap | 02125366 |
| pms-Hydromorphone 2mg Tab | 00885436 | Jurnista 16mg Tab | 02337282 |
| Teva-Hydromorphone 2mg Tab | 02319411 | Hydromorph Contin 18mg Cap | 02243562 |
| Dilaudid 2mg Tab | 00125083 | Hydromorphone HP 20mg/mL Inj | 02145936 |
| Hydromorph Contin 3mg Cap | 02125323 | Hydromorph Contin 24mg Cap | 02125382 |
| Apo-Hydromorphone 4mg Tab | 02364131 | Hydromorph Contin 30mg Cap | 02125390 |
| pms-Hydromorphone 4mg Tab | 00885401 | Jurnista 32mg Tab | 02337290 |
| Teva-Hydromorphone 4mg Tab | 02319438 | Hydromorphone HP 50mg/mL Inj | 02146126 |
| Dilaudid 4mg Tab | 00125121 | Hydromorph Contin 4.5mg Cap | 02359502 |
| Jurnista 4mg Tab | 02337266 | Hydromorph Contin 6mg Cap | 02125331 |
| **Oxycodone** | | | |
| Targin 2.5/5mg Tab | 02387425 | Targin 10/20mg Tab | 02339617 |
| pms-Oxycodone 5mg Tab | 02319977 | OxyNeo 15mg Tab | 02372533 |
| Oxy-IR 5mg Tab | 02231934 | pms-Oxycodone 20mg Tab | 02319993 |
| pms-Oxycodone 5mg Tab | 02319977 | Oxy-IR 20mg Tab | 02240132 |
| Supeudol 5mg Tab | 00789739 | pms-Oxycodone 20mg Tab | 02319993 |
| Targin 5/10mg Tab | 02339609 | Supeudol 20mg Tab | 02262983 |
| pms-Oxycodone 10mg Tab | 02319985 | OxyNeo 20mg Tab | 02372797 |
| Oxy-IR 10mg Tab | 02240131 | Supeudol 20mg Supp | 00392472 |
| pms-Oxycodone 10mg Tab | 02319985 | Targin 20/40mg Tab | 02339625 |
| Supeudol 10mg Tab | 00443948 | OxyNeo 30mg Tab | 02372541 |
| OxyNeo 10mg Tab | 02372525 | OxyNeo 40mg Tab | 02372568 |
| Supeudol 10mg Supp | 00392480 | OxyNeo 60mg Tab | 02372576 |
|  |  | OxyNeo 80mg Tab | 02372584 |
| **Codeine, combination excluding psycholeptics** | | | |
| ratio-Emtec Tab | 00608882 | Atasol 30 Tab | 00293512 |
| ratio-Lenoltec #3 Tab | 00653276 | Tylenol #3 Tab | 02163926 |
| ratio-Lenoltec #4 Tab | 00621463 | Tylenol #4 Tab | 02163918 |
| **Meperidine** | | | |
| Meperidine 50mg/mL Inj | 00725765 | Meperidine 75mg/mL Inj | 00725757 |
| Demerol 50mg Tab | 02138018 | Meperidine 100mg/mL Inj | 00725749 |
| **Fentanyl** | | | |
| CO Fentanyl 12mcg/hr Patch | 02386844 | RAN-Fentanyl MTX 75mcg/hr Patch | 02330148 |
| MYLAN-Fentanyl Matrix 12mcg/hr Patch | 02396696 | Sandoz Fentanyl 75mcg/hr Patch | 02327155 |
| pms-Fentanyl MTX 12mcg/hr Patch | 02341379 | Teva-Fentanyl 75mcg/hr Patch | 02282976 |
| RAN-Fentanyl MTX 12mcg/hr Patch | 02330105 | Duragesic MAT 75mcg/hr Patch | 02275848 |
| Sandoz Fentanyl 12mcg/hr Patch | 02327112 | Apo-Fentanyl 100mcg/hr Patch | 02314665 |
| Teva-Fentanyl 12mcg/hr Patch | 02311925 | CO Fentanyl 100mcg/hr Patch | 02386895 |
| Apo-Fentanyl 25mcg/hr Patch | 02314630 | MYLAN-Fentanyl Matrix 100mcg/hr Patch | 02396742 |
| CO Fentanyl 25mcg/hr Patch | 02386852 | pms-Fentanyl 100mcg/hr Patch | 02341417 |
| MYLAN-Fentanyl Matrix 25mcg/hr Patch | 02396718 | RAN-Fentanyl MTX 100mcg/hr Patch | 02330156 |
| pms-Fentanyl MTX 25mcg/hr Patch | 02341387 | Sandoz Fentanyl 100mcg/hr Patch | 02327163 |
| RAN-Fentanyl MTX 25mcg/hr Patch | 02330113 | Teva-Fentanyl 100mcg/hr Patch | 02282984 |
| Sandoz Fentanyl 25mcg/hr Patch | 02327120 | Duragesic MAT 100mcg/hr Patch | 02275856 |
| Teva-Fentanyl 25mcg/hr Patch | 02282941 | Abstral 100mcg SL Tab | 02364174 |
| Duragesic MAT 25mcg/hr Patch | 02275813 | Abstral 200mcg SL Tab | 02364182 |
| Apo-Fentanyl 50mcg/hr Patch | 02314649 | Abstral 300mcg SL Tab | 02364190 |
| CO Fentanyl 50mcg/hr Patch | 02386879 | Abstral 400mcg SL Tab | 02364204 |
| MYLAN-Fentanyl Matrix 50mcg/hr Patch | 02396726 | Abstral 600mcg SL Tab | 02364212 |
| pms-Fentanyl 50mcg/hr Patch | 02341395 | Fentanyl Citrate 50mcg/mL Inj | 00888346 |
| RAN-Fentanyl MTX 50mcg/hr Patch | 02330121 | Fentanyl Citrate 50mcg/mL Inj | 02240434 |
| Sandoz Fentanyl 50mcg/hr Patch | 02327147 | CO Fentanyl 75mcg/hr Patch | 02386887 |
| Teva-Fentanyl 50mcg/hr Patch | 02282968 | MYLAN-Fentanyl Matrix 75mcg/hr Patch | 02396734 |
| Duragesic MAT 50mcg/hr Patch | 02275821 | pms-Fentanyl 75mcg/hr Patch | 02341409 |
| Apo-Fentanyl 75mcg/hr Patch | 02314657 |  |  |
| **Sufentanil** | | | |
| Sufentanil Citrate 50mcg/mL Inj | 02244147 | Sufentanil Citrate 50mcg/mL Inj | 02442213 |
| **Ketamine** | | | |
| Ketamine 10mg/mL Inj | 02246795 | Ketamine 50mg/mL Inj | 02246796 |
| Ketalar 10mg/mL Inj | 00224391 | Ketalar 50mg/mL Inj | 00224405 |
| **Dextropropoxyphene** | | | |
| Talwin 30mg/mL Inj | 02241976 | Talwin 50mg Tab | 02137984 |
| **Buprenorphine*** | | | |
| Butrans-5 5mcg/hr Patch * | 02341174 | Butrans-20 20mcg/hr Patch * | 02341220 |
| Butrans-10 10mcg/hr Patch * | 02341212 |  |  |
| **Butorphanol** | | | |
| Apo-Butorphanol Nasal Sp | 02242504 |  |  |
| **Tramadol** | | | |
| Apo-Tramadol 50mg Tab | 02426153 | Ralivia 200mg Tab | 02299208 |
| Ultram 50mg Tab | 02349469 | Zytram XL 200mg Tab | 02286432 |
| Taro-Tramadol ER 100mg Tab | 02450429 | Taro-Tramadol ER 300mg Tab | 02450445 |
| Tridural 100mg Tab | 02296381 | Tridural 300mg Tab | 02296411 |
| Ralivia 100mg Tab | 02299194 | Ralivia 300mg Tab | 02299216 |
| Zytram XL 150mg Tab | 02286424 | Zytram XL 300mg Tab | 02286440 |
| Taro-Tramadol ER 200mg Tab | 02450437 | Zytram XL 400mg Tab | 02286459 |
| Tridural 200mg Tab | 02296403 |  |  |
| **Tramadol (combinations)** | | | |
| Apo-Tramadol/Acet 37.5/325mg Tab | 02336790 | pms-Tramadol/Acet 37.5/325mg Tab | 02401657 |
| Auro-Tramadol/Acetaminop 37.5mg/325mg Tab | 02439050 | RAN-Tramadol/Acet 37.5/325mg Tab | 02388197 |
| CO Tramadol/Acet 37.5/325mg Tab | 02383209 | Teva-Tramadol/Acetaminophen 37.5/325mg Tab | 02347180 |
| Jamp-Acet-Tramadol 325/37.5mg Tab | 02388308 | Tramadol/Acet 37.5/325mg Tab | 02426803 |
| Mar-Tramadol/Acet 37.5/325mg Tab | 02388324 | Tramadol/Acet 37.5/325mg Tab | 02429969 |
| MINT-Tramadol/Acet 37.5/325mg Tab | 02389800 | Tramacet 37.5/325mg Tab | 02264846 |
| **Methadone*** | | | |
| Metadol 1mg Tab | 02247698 | Metadol 10mg Tab | 02247700 |
| Metadol 1mg/mL O/L | 02247694 | Metadol 10mg/mL O/L | 02241377 |
| Metadol 5mg Tab | 02247699 | Metadol 25mg Tab | 02247701 |
| **Additional DINs (Not identified in formulary)** | | | |
| 282 Mep Tab | | | 00002238646 |
| 282 Tablets | | | 00002234510 |
| 292 Tab | | | 00000219843 |
| 292 Tab 375mg | | | 00002238645 |
| Ac & C Tab 8mg | | | 00000180041 |
| Acetaminophen 300mg With Caffeine & Codeine Cap | | | 00002154234 |
| Acetaminophen Caffeine & 8mg Cod.Phos. Tab | | | 00001997688 |
| Acetaminophen Compound Caplets With Codeine | | | 00002028174 |
| Acetaminophen Compound Tab With Codeine | | | 00002025337 |
| Acetaminophen W Caffeine & Codeine Tab 8mg | | | 00000706221 |
| Acetaminophen With Codeine - Caplet 300mg | | | 00002143933 |
| Acetaminophen, Caffeine & Codeine Tab 8mg | | | 00002251914 |
| Acetazone Forte C-8 Tab | | | 00000834319 |
| Acet-Codeine Tab 30mg | | | 00001999648 |
| Act Buprenorphine/Naloxone Tab 2mg/.5mg | | | 00002453908 |
| Act Buprenorphine/Naloxone Tab 8mg/2mg | | | 00002453916 |
| Act Oxycodone Cr Extended Release Tab 10mg | | | 00002394189 |
| Act Oxycodone Cr Extended Release Tab 20mg | | | 00002394197 |
| Act Oxycodone Cr Extended Release Tab 40mg | | | 00002394200 |
| Act Oxycodone Cr Extended Release Tab 5mg | | | 00002394170 |
| Act Oxycodone Cr Extended Release Tab 80mg | | | 00002394219 |
| Apo-Oxycodone Cr Extended Release Tab 10mg | | | 00002366754 |
| Apo-Oxycodone Cr Extended Release Tab 15mg | | | 00002394766 |
| Apo-Oxycodone Cr Extended Release Tab 20mg | | | 00002366762 |
| Apo-Oxycodone Cr Extended Release Tab 30mg | | | 00002394774 |
| Apo-Oxycodone Cr Extended Release Tab 40mg | | | 00002306530 |
| Apo-Oxycodone Cr Extended Release Tab 5mg | | | 00002366746 |
| Apo-Oxycodone Cr Extended Release Tab 60mg | | | 00002394782 |
| Apo-Oxycodone Cr Extended Release Tab 80mg | | | 00002366789 |
| Apo-Oxycodone/Acet Tab 5/325mg | | | 00002324628 |
| Atasol-15 Tab 300mg | | | 00000293504 |
| Atasol-8 Tab | | | 00000293490 |
| Belbuca Soluble Film 150mcg/Dose* | | | 00002465248 |
| Belbuca Soluble Film 75mcg/Dose* | | | 00002465221 |
| Butrans 15 Patch 15mcg/Hour* | | | 00002450771 |
| Calmylin Codeine Syrup 10mg/5ml** | | | 00002172917 |
| Calmylin Ace Codeine Syrup 10mg/5ml** | | | 00002198630 |
| Calmylin W Codeine Cough Syr 19.8mg/30ml Original** | | | 00000535230 |
| Coactifed (Expectorant) Liq 10mg/5ml** | | | 00000068756 |
| Coactifed Syrup 10mg/5ml** | | | 00000068594 |
| Coactifed Tab 20mg** | | | 00000068608 |
| Codeine Contin Sustained-Release Tab 50mg | | | 00002230302 |
| Codeine Contin Tab 100mg | | | 00002163748 |
| Codeine Contin Tab 150mg | | | 00002163780 |
| Codeine Contin Tab 200mg | | | 00002163799 |
| Codeine Phosphate Liq Inj Usp 30mg/Ml | | | 00000544884 |
| Codeine Phosphate Pdr | | | 00000905518 |
| Codeine Phosphate Syrup 4.7666mg | | | 00000050024 |
| Codeine Powder | | | 00099099975 |
| Codeine Syrup 5mg/Ml | | | 00000093114 |
| Codeine Tab 30mg | | | 00000779466 |
| Codeine Tab 30mg | | | 00000093130 |
| Codeine Tab 30mg | | | 00002009757 |
| Cophylac** | | | 00001987577 |
| Cophylac** | | | 00002224577 |
| Cophylac Dps** | | | 00000116343 |
| Darvon N 100mg Cap | | | 00000261432 |
| Demerol Hcl Tab 50mg | | | 00000033685 |
| Demerol Inj 100mg/Ml Liq | | | 00002242005 |
| Demerol Inj 50mg/Ml Liq | | | 00002139022 |
| Dilaudid Hp Plus Inj 20mg/Ml Liq | | | 00002146118 |
| Dilaudid Sup 3mg | | | 00000125105 |
| Dilaudid Tab 2mg | | | 00000290572 |
| Dilaudid Tab 4mg | | | 00000290602 |
| Dimetane Expectorant C Syr 10mg/5ml** | | | 00002244079 |
| Dimetane Expectorant C Syr 10mg/5ml** | | | 00001934716 |
| Dimetane Expectorant Dc Syr** | | | 00001934708 |
| Dimetane Expectorant Dc Syrup 20mg/Ml** | | | 00002244080 |
| Dimetapp C Syr** | | | 00002244078 |
| Doloral 5 Sirop 5mg/Ml | | | 00000614505 |
| Duragesic Mat Patch 12mcg/Hr | | | 00002334186 |
| Duragesic Patch 12mcg/Hour | | | 00002280345 |
| Duragesic Patch Srd 100mcg/Hr | | | 00001937413 |
| Duragesic Patch Srd 25mcg/Hr | | | 00001937383 |
| Duragesic Patch Srd 50mcg/Hr | | | 00001937391 |
| Duragesic Patch Srd 75mcg/Hr | | | 00001937405 |
| Endocet Tab 325/5 Mg | | | 00000574384 |
| Endocet Tab 5/325 Mg | | | 00001916548 |
| Fentanyl Citrate Inj Liq | | | 00002384124 |
| Fentanyl Citrate Inj Liq 50mcg/Ml | | | 00002385406 |
| Fentanyl Compound | | | 00000994025 |
| Fentanyl Patch Srd 50mcg/Hr | | | 00002304139 |
| Fentora Tab 100mcg | | | 00002408007 |
| Fentora Tab 200mcg | | | 00002408015 |
| Fentora Tab 400mcg | | | 00002408023 |
| Fentora Tab 600mcg | | | 00002408031 |
| Fiorinal C 1/2 Cap | | | 00000176206 |
| Fiorinal C 1/4 Cap | | | 00000176192 |
| Hycodan Syrup 1mg/Ml** | | | 00001916580 |
| Hycodan Tab 5mg** | | | 00001916599 |
| Hydromorph Ir Tab 2mg | | | 00002245703 |
| Hydromorph Ir Tab 4mg | | | 00002245704 |
| Hydromorphone Compound | | | 00000994006 |
| Hydromorphone Hcl Liq 10mg | | | 00002460610 |
| Hydromorphone Hcl Liq Inj 2mg/Ml | | | 00002460602 |
| Hydromorphone Hcl Usp High Potency 10mg | | | 00002382636 |
| Hydromorphone Hp Forte Inj 100mg/Ml Liq | | | 00002244797 |
| Hydromorphone Powder | | | 00099099980 |
| Lomotil Tab 2.5mg** | | | 00000399345 |
| Lomotil Tab 2.5mg** | | | 00000036323 |
| M.O.S. "1" Syr 1mg/Ml | | | 00000486582 |
| M.O.S. "10" Tab 10mg | | | 00000690198 |
| M.O.S. "20" Conc Liq 20mg/Ml | | | 00000632481 |
| M.O.S. "20" Tab 20mg | | | 00000690201 |
| M.O.S. "5" Syr 5mg/Ml | | | 00000514217 |
| M.O.S. "60" Tab 60mg | | | 00000690244 |
| M.O.S. Sr Tab 30mg | | | 00000776181 |
| M.O.S. Sr Tab 60mg | | | 00000776203 |
| M.O.S. Sulphate Tab 10mg | | | 00002009765 |
| M.O.S. Sulphate Tab 25mg | | | 00002009749 |
| M.O.S. Sulphate Tab 50mg | | | 00002009706 |
| M.O.S. Sulphate Tab 5mg | | | 00002009773 |
| Meperidine Hcl Liq Inj 100mg/Ml | | | 00000497479 |
| Mersyndol Tab | | | 00002047667 |
| M-Eslon Ir Cap 10mg | | | 00002320428 |
| M-Eslon Ir Cap 30mg | | | 00002320444 |
| Metadol-D Oral 10mg/Ml | | | 00002244290 |
| Methadone Injectable | | | 00000994901 |
| Methadone O/L (Mg) | | | 00099099993 |
| Methadone Pwd (Compound) 1mg | | | 00000999734 |
| Methadose Sol'n 10mg/Ml | | | 00002394596 |
| Methadose Unflavored Sol'n 10mg/Ml | | | 00002394618 |
| Methoxacet C 1/8 Caplets | | | 00002236872 |
| Methoxisal C 1/4 | | | 00001966367 |
| Methoxisal C1/2 Caplets | | | 00001966375 |
| Morphine Hp 25 Liq Inj 25mg/Ml | | | 00000676411 |
| Morphine Hydrochloride Compound | | | 00000994009 |
| Morphine Liq Inj 10mg/Ml | | | 00002382997 |
| Morphine Powder | | | 00099099986 |
| Morphine Sr Tab 100mg | | | 00002350920 |
| Morphine Sr Tab 200mg | | | 00002350947 |
| Morphine Sulfate Liq Inj 10mg/Ml | | | 00000850322 |
| Morphine Sulfate Liq Inj 10mg/Ml | | | 00000497355 |
| Morphine Sulfate Liq Inj 5mg/Ml Usp Iv | | | 00000649619 |
| Morphine Sulphate Pws | | | 00000999955 |
| Muscle & Back Pain Relief - 8 Tab | | | 00002242180 |
| Mylan-Buprenorphine/Naloxone Tab 2mg/.5mg* | | | 00002408090 |
| Mylan-Buprenorphine/Naloxone Tab 8mg/2mg* | | | 00002408104 |
| Nf Cough Syrup With Codeine** | | | 00002099748 |
| Novahistex Dh Adult Syrup** | | | 00002049481 |
| Novahistine Dh Children Syr** | | | 00002049473 |
| Nucynta Cr Tab 100mg | | | 00002360381 |
| Nucynta Cr Tab 150mg | | | 00002360403 |
| Nucynta Cr Tab 200mg | | | 00002360411 |
| Nucynta Cr Tab 50mg | | | 00002360373 |
| Nucynta Extended Release Tab 100mg | | | 00002415585 |
| Nucynta Extended Release Tab 150mg | | | 00002415593 |
| Nucynta Extended Release Tab 200mg | | | 00002415607 |
| Nucynta Extended Release Tab 250mg | | | 00002415615 |
| Nucynta Extended Release Tab 50mg | | | 00002415577 |
| Nucynta Ir Tab 100mg | | | 00002378299 |
| Nucynta Ir Tab 50mg | | | 00002378272 |
| Nucynta Ir Tab 75mg | | | 00002378280 |
| Onsolis Soluble Film 200mcg | | | 00002350661 |
| Onsolis Soluble Film 400mcg | | | 00002350688 |
| Onsolis Soluble Film 600mcg | | | 00002350696 |
| Onsolis Soluble Film 800mcg | | | 00002350718 |
| Opium & Belladona Sup | | | 00001923463 |
| Oxycodone Tab 5mg | | | 00002325950 |
| Oxycodone/Acet Tab 5/325mg | | | 00002361361 |
| Oxycodone-Acet Tab 5/325mg | | | 00002327171 |
| Oxycontin Extended Release Tab 15mg | | | 00002323192 |
| Oxycontin Extended Release Tab 30mg | | | 00002323206 |
| Oxycontin Extended Release Tab 60mg | | | 00002323214 |
| Oxycontin Srt 10mg | | | 00002202441 |
| Oxycontin Srt 20mg | | | 00002202468 |
| Oxycontin Srt 40mg | | | 00002202476 |
| Oxycontin Srt 5mg | | | 00002258129 |
| Oxycontin Srt 80mg | | | 00002202484 |
| Pat-Fentanyl Mat Patch 12mcg/Hour | | | 00002376768 |
| Percocet Demi Tab 2.5mg/325mg | | | 00001916491 |
| Percocet Tab 5/325 Mg | | | 00000389641 |
| Percocet Tab 5mg/325 Mg | | | 00001916475 |
| Percodan 325/5mg Tab | | | 00001916572 |
| Pharmasave Cough Syr | | | 00000690074 |
| Phl-Hydromorphone Tab 2mg | | | 00002249928 |
| Pms-Acetaminophen W Codeine Elixir 32mg | | | 00000816027 |
| Pms-Buprenorphine/Naloxone Sublingual Tab 8/2mg* | | | 00002424878 |
| Pms-Buprenorphine/Naloxone Sublingual Tab 2/.5mg* | | | 00002424851 |
| Pms-Butorphanol Nasal Spray 10mg/Ml | | | 00002244508 |
| Pms-Codeine 15mg Tab | | | 00002243978 |
| Pms-Codeine 30mg Tab | | | 00002243979 |
| Pms-Hydrocodone Syr 1mg/Ml | | | 00002324253 |
| Pms-Hydromorphone Supp 3mg | | | 00001916394 |
| Pms-Morphine Sulfate Srt 15mg | | | 00002245284 |
| Pms-Morphine Sulfate Srt 30mg | | | 00002245285 |
| Pms-Morphine Sulfate Srt 60mg | | | 00002245286 |
| Pms-Morphine Sulfate Srt 100mg | | | 00002245287 |
| Pms-Morphine Sulfate Srt 200 Mg | | | 00002245288 |
| Pms-Opium And Belladona Sup | | | 00000815349 |
| Pms-Oxycodone Cr Extended Release Tab 10mg | | | 00002309882 |
| Pms-Oxycodone Cr Extended Release Tab 20mg | | | 00002309890 |
| Pms-Oxycodone Cr Extended Release Tab 40mg | | | 00002309904 |
| Pms-Oxycodone Cr Extended Release Tab 80mg | | | 00002309912 |
| Pms-Oxycodone-Acetaminophen 325/5mg | | | 00002245758 |
| Ran-Fentanyl Transdermal 100mcg/Hour | | | 00002249448 |
| Ran-Fentanyl Transdermal 25mcg/Hour | | | 00002249391 |
| Ran-Fentanyl Transdermal 50mcg/Hour | | | 00002249413 |
| Ran-Fentanyl Transdermal 75mcg/Hour | | | 00002249421 |
| Ratio-Codeine Syrup 5mg/Ml | | | 00000779474 |
| Ratio-Codeine Tab 15mg | | | 00000593435 |
| Ratio-Codeine Tab 30mg | | | 00000593451 |
| Ratio-Cotridin 10mg/5ml | | | 00002169126 |
| Ratio-Lenoltec #1 Tab | | | 00000653233 |
| Ratio-Lenoltec #2 Tab 300mg | | | 00000653241 |
| Ratio-Morphine Syrup 10mg/Ml | | | 00000690783 |
| Ratio-Morphine Syrup 1mg/Ml | | | 00000607762 |
| Ratio-Morphine Syrup 5mg/Ml | | | 00000607770 |
| Ratio-Morphine Syrup Conc 20mg/Ml | | | 00000690791 |
| Ratio-Oxycocet Tab 5/325 Mg | | | 00000608165 |
| Ratio-Oxycodan Tab 5mg/325mg | | | 00000608157 |
| Ratio-Tecnal C 1/2 Cap | | | 00000608181 |
| Ratio-Tecnal-C 1/4 Cap | | | 00000608203 |
| Robaxacet-8 Tab | | | 00001934767 |
| Robaxisal C-1/2 Tab | | | 00001934791 |
| Robaxisal C-1/4 Tab | | | 00001934783 |
| Robitussin A-C Syr 10mg/5ml** | | | 00001934740 |
| Sandoz Fentanyl Patch 37mcg/H | | | 00002327139 |
| Sandoz Opium & Belladonna Sup | | | 00001901869 |
| Sandoz Oxycodone/Acetaminophen Tab 5mg/325mg | | | 00002307898 |
| Statex Supp 5mg | | | 00000632228 |
| Suboxone Sublingual Tab 12/3mg* | | | 00002468085 |
| Suboxone Sublingual Tab 2/.5mg* | | | 00002295695 |
| Suboxone Sublingual Tab 8/2mg* | | | 00002295709 |
| Tussionex Srt | | | 00001916963 |
| Tussionex Suspension Srs | | | 00001916971 |
| Tylenol No.1 Caplets | | | 00002181061 |
| Tylenol No.2 Tab 300mg | | | 00002163934 |
| Tylenol No.3 Tab | | | 00000425389 |
| Tylenol WITH CODEINE ELIXIR 32MG | | | 00002163942 |

*Buprenorphine and methadone formulations excluded after initial identification as presented in Figure 1

** Cough and anti-diarrhea medications excluded after initial identification as presented in Figure 1

**Appendix 3: ED visit and procedure codes that were used to identify subjects who had procedures or ED visits on or up to 14 days from the opioid fill.**  Any individual with a documented procedure code from the list below or a code for an ED visit occurring on or up to 14 days prior to an opioid fill was eligible for inclusion in the cohort. The choice of 14 days postoperatively aligns with studies that have considered opioid fills occurring within 14 days of surgery to be within the “peri-operative period” (i.e., directly related to the surgery).^21,22^ We used a broad definition of surgical procedures that included all Canadian Classification of Procedures (CCP) and Canadian Classification of Health Interventions (CCI) codes, which include obstetrical care and some interventional procedures that are carried out by internal medicine specialties.

| **Data source** | **Variables used** | **Description** | **Categorization** | **Code category/ range** |
| --- | --- | --- | --- | --- |
| MSI Physicians’ Billings | ccpcat (CCP Category) | Category of the procedure for which the individual was billed | (Bone Grafts) | BOGR |
|  |  |  | (Casts and Splints) | CASP |
|  |  |  | (Dislocation) | DISL |
|  |  |  | (Major Fracture) | MAFR |
|  |  |  | (Major Surgery) | MASG |
|  |  |  | (Minor Fracture) | MIFR |
|  |  |  | (Minor Surgery) | MISG |
| CIHI DAD | Intervention code 11/02 (CCI code) | Category for the intervention which the individual had received | (Obstetrical) | OBST |
|  |  |  | Therapeutic Interventions on the Eye and Ocular Adnexa | 1CC - 1CZ |
|  |  |  | Therapeutic Interventions on the Ear and Mastoid (process) | 1DA - 1DZ |
|  |  |  | Therapeutic Interventions on the Orocraniofacial Region | 1EA - 1FX |
|  |  |  | Therapeutic Interventions on the Respiratory System | 1GA - 1GZ |
|  |  |  | Therapeutic Interventions on the Cardiovascular System | 1HA - 1LZ |
|  |  |  | Therapeutic Interventions on the Lymphatic System | 1MA - 1MZ |
|  |  |  | Therapeutic Interventions on the Digestive and Hepatobiliary Tracts and Other Sites within the Abdominal Cavity NEC | 1NA - 1OZ |
|  |  |  | Therapeutic Interventions on the Genitourinary System | 1PB - 1RZ |
|  |  |  | Therapeutic Interventions on the Musculoskeletal System | 1SA - 1WZ |
|  |  |  | Therapeutic Interventions on the Skin, Subcutaneous Tissue and Breast | 1YA - 1YZ |
|  |  |  | Antepartum Interventions | 5AB - 5CA |
|  |  |  | Interventions During Labour and Delivery | 5LB - 5MD |
| NACRS | ED visit (leaving ED), derived from [registration_date] | A date representing leaving the ED | Yes vs. No | NA |

**Appendix 4: Dose calculation and opioid variable definitions.**

**Table: MME calculation formula, oral opioid analgesic conversion factors. (**Source: Ontario Drug Policy Research Network. ODPRN suggested calculation of opioid milligrams of morphine equivalents. Toronto: Ontario Drug Policy Research Network; November 2020.)

| **Opioid** | **Number Mg** | **Ratio (Opioid : Morphine)** |
| --- | --- | --- |
| Morphine | 30 mg | 1:1 |
| Codeine | 200 mg | 1:0.15 |
| Oxycodone | 15-20 mg | 1:1.5 |
| Hydrocodone | 30 mg | 1:1 |
| Hydromorphone | 6-7.5 mg | 1:5 |
| Meperidine | 300 mg | 1:0.1 |
| Tramadol | 300 mg | 1:0.1 |

We used the following formula to calculate dose: MME=quantity * strength * conversion factor. We used the conversion factors as described in the Table, and divided the total MME by days’ supply to estimate the average daily MME. Only opioids taken through the oral route were included in dose calculations. For subjects with multiple opioid fills on index day, total MME was summed across all filled prescriptions and divided by the number of days of the prescriptions with the longest days’ supply. Long-lasting formulations were prioritized in the opioid type variable when both short- and long-acting formulations were filled.

We decided post-hoc to truncate days’ supply at 30 days and MME/day at 200 MME. The number of excluded subjects who exceeded these thresholds were 95 and 128 (less than 0.01% of study population) for dose and MME, respectively. These decisions had no impact on study outcomes.

**Definition of short and long-acting opioids:** All formulations that were labeled as extended-release, sustained-release, or long-acting were considered long-acting opioids, while all other formulations were considered short-acting.

**Definition of weak and strong opioids:** Hydromorphone, oxycodone, morphine, and fentanyl were considered strong opioids, while codeine and tramadol were considered weak.

**Definition of opioid use disorder medications (used for exclusion):** All buprenorphine and methadone formulations.

**Appendix 5: ICD diagnostic codes used to measure 12-month history of physical and mental health co-morbidities.** For each of the included health conditions, we defined history of conditions as present if at least one of the corresponding diagnostic codes was found in MSI Physicians’ Billings (ICD-9-CM) or CIHI DAD (ICD-10-CA) databases in the 12 months prior to the index fill. Studies previously applying variable definition are cited.

| Variable | ICD-9-CM codes* | ICD-9-CM code labels | ICD-10-CA codes* | ICD-10-CA code labels |
| --- | --- | --- | --- | --- |
| History of depression | 296.2, 296.3, 296.5, 300.4, 309, 311 (MCHP, Himelhoch 2004) | 296.2 Major depressive disorder, single episode  296.3, Major depressive disorder, recurrent episode  296.5, Bipolar I disorder, most recent episode (or recurrent) depressed  300.4, Dysthymic disorder  309, Adjustment reaction  311 Depressive disorder NEC | F20.4, F31.3–F31.5, F32, F33, F34.1, F41.2, F43.2 (MCHP) | F20.4, Post-schizophrenic depression  F31.3–F31.5, Bipolar affective disorder, current episode mild or moderate depression; current episode severe depression without psychotic symptoms; current episode severe depression with psychotic symptoms  F32, Depressive episode  F33, Recurrent depressive disorder  F34.1, Dysthymia  F41.2, Mixed anxiety and depressive disorder  F43.2, Adjustment disorders |
| History of anxiety disorder | 300 (ICD Manual) | Anxiety, dissociative and somatoform disorders | F32.0, F40, F41, F42, F44, F45.0, F45.1, F45.2, F48, F68.0, F99 (Martens 2015) | F32.0, Mild depressive episode  F40, Phobic anxiety disorders  F41, Other anxiety disorders  F42, Obsessive-compulsive disorder  F44, Dissociative [conversion] disorders  F45.0, Somatization disorder  F45.1, Undifferentiated somatoform disorder  F45.2, Hypochondriacal disorder  F48, Other neurotic disorders  F68.0, Other disorders of adult personality and behavior  F99 Mental disorder, not otherwise specified |
| History of alcohol abuse | 265.2, 291.1–291.3, 291.5, 291.8, 291.9, 303.0, 303.9, 305.0, 357.5, 425.5, 535.3, 571.0, 571.1–571.3, 980, V11.3 (MCHP) | 265.2 Pellagra,  291.1–291.3 Alcoholic amnestic disorder, alcohol persist dementia, alcohol-induced psychotic disorder with hallucinations  291.5 Alcohol-induced psychotic disorder with delusions  291.8, Other specified alcohol-induced mental disorders  291.9, Unspecified alcohol-induced mental disorders  303.0, Acute alcoholic intoxication  303.9, Other and unspecified alcohol dependence  305.0, Alcohol abuse  357.5, Alcoholic polyneuropathy  425.5, Alcoholic cardiomyopathy  535.3, Alcoholic gastritis  571.0, Alcoholic fatty liver  571.1–571.3, Acute alcoholic hepatitis; alcoholic cirrhosis of liver; alcoholic liver damage, unspecified  980, Toxic effect of alcohol  V11.3 Personal history of alcoholism | E52, F10, G62.1, I42.6, K29.2, K70.0, K70.3, K70.9, T51, Z50.2, Z71.4, Z72.1  (MCHP), Z86.40 (ICD Manual) | History of Alcohol Abuse  E52, Niacin deficiency [pellagra]  F10, Mental and behavioural disorders due to use of alcohol  G62.1, Alcoholic polyneuropathy  I42.6, Alcoholic cardiomyopathy  K29.2, Alcoholic gastritis  K70.0, Alcoholic fatty liver  K70.3, Alcohol cirrhosis of liver  K70.9, Alcoholic liver disease, unspecified  T51, Toxic effect of alcohol  Z50.2, Alcohol rehabilitation  Z71.4, Alcohol abuse counselling and surveillance  Z72.1, Alcohol use  Z86.40 Personal history of alcohol abuse |
| History of drug and substance abuse | 292, 304, 305.2–305.9, V65.42 (MCHP) | 292, Drug-induced mental disorders  304, Drug dependence  305.2–305.9, Cannabis abuse; hallucinogen abuse; sedative, hypnotic or anxiolytic abuse; opioid abuse; cocaine abuse; amphetamine or related acting sympathomimetic abuse; antidepressant type abuse; other, mixed, or unspecified drug abuse  V65.42Counseling on substance use and abuse | F16, F18, F19, Z71.5, Z72.2 (MCHP), Z50.3, Z86.41, Z86.48 (ICD Manual) | F16, Mental and behavioural disorders due to use of hallucinogens  F18, Mental and behavioural disorders due to use of volatile solvents  F19, Mental and behavioural disorders due to multiple drug use and use of other psychoactive substances  Z71.5, Drug abuse counselling and surveillance  Z72.2, Drug use  Z50.3, Drug rehabilitation  Z86.41, Personal history of drug abuse  Z86.48, Personal history of other psychoactive substance abuse |
| History of tobacco abuse | 305.1, V15.82 (ICD Manual) | 305.1, Tobacco use disorder  V15.82 History of tobacco use | F17, Z71.6, Z72.0, Z86.42, T65.2 (ICD Manual) | F17, Mental and behavioural disorders due to use of tobacco  Z71.6, Tobacco abuse counselling  Z72.0, Tobacco use  Z86.42, Personal history of tobacco abuse  T65.2 (ICD Manual) Toxic effect of tobacco and nicotine |
| History of low back pain | 721.3x – 721.9x, 722.2x, 722.30, 722.70, 722.80, 722.90, 722.32, 722.72, 722.82, 722.92, 722.73, 722.83, 722.93, 724.xx, 737.1, 737.3, 738.4, 738.5, 739.2, 739.3, 739.4, 756.10, 756.11, 756.12, 756. 13, 756.19, 805.4, 805.8, 839.2, 839.42, 846, 846.0, 847.1, 847.3, 847.2, 847.9 (Shah 2017) | 721.3x – 721.9x, Lumbosacral spondylosis without myelopathy; thoracic or lumbar spondylosis with myelopathy;  722.2x, Displacement of intervertebral disc, site unspecified, without myelopathy  722.30, Schmorl’s nodes, unspecified region  722.70, Intervertebral disc disorder with myelopathy, unspecified region  722.80, Postlaminectomy syndrome, unspecified region  722.90, Other and unspecified disc disorder, unspecified region  722.32, Schmorl’s nodes, lumbar region  722.72, Intervertebral disc disorder with myelopathy, thoracic region  722.82, Postlaminectomy syndrome, thoracic region  722.92, Other and unspecified disc disorder, thoracic region  722.73, Intervertebral disc disorder with myelopathy, lumbar region  722.83, Postlaminectomy syndrome, lumbar region  722.93, Other and unspecified disc disorder, lumbar region  724.xx, Other and unspecified disorders of back  737.1, Kyphosis (acquired)  737.3, Kyphoscoliosis and scoliosis  738.4, Acquired spondylolisthesis  738.5, Other acquired deformity of back or spine  739.2, Nonallopathic lesions, thoracic region  739.3, Nonallopathic lesions, lumbar region  739.4, Nonallopathic lesions, sacral region  756.10, Anomaly of spine NOS  756.11, Lumbosacral spondylolysis  756.12, Spondylolisthesis  756.13, Absence of vertebra, congenital  756.19, Other anomalies of spine  805.4, Closed fracture of lumbar vertebra without mention of spinal cord injury  805.8, Closed fracture of unspecified vertebral column without mention of spinal cord injury  839.2, Dislocation; thoracic and lumbar vertebra, closed  839.42, Closed dislocation, sacrum  846, Sprains and strains of sacroiliac region  846.0, Sprain lumbosacral  847.1, Sprain thoracic region  847.3, Sprain of sacrum  847.2, Sprain lumbar region  847.9, Sprain of back NOS | M51.3, M54.3, M54.4, M54.5, M54.8, M54.9, (HQ Ontario) | M51.3, Other specified intervertebral disc degeneration  M54.3, Sciatica  M54.4, Lumbago with sciatica  M54.5, Low back pain  M54.8, Other dorsalgia  M54.9, Dorsalgia, unspecified site |
| History of headache/migraine | >=346 AND <347 OR 307.81 (Shah 2017) | >=346 AND <347 Migraine; 307.81 tension headache | G43-G44, R51 (ICD Manual) | G43-G44, Migraine, Other headache syndromes  R51 Headache |
| History of arthritis/joint pain or neck pain | >=710 AND <720 OR >=725 AND <740 (Shah 2017)  721.0x, 721.1x, 722.0x, 722.31, 722.71, 722.81, 722.91, 723.XX, 839.0, 839.1, 847. 0 (Shah 2017) | >=710 AND <720 (710 to 719) Arthropathies and related disorder  OR  >=725 AND <740  (725 to 729) Rheumatism, excluding the back  (730 to 739) Osteopathies, chondropathies, and acquired musculoskeletal deformities  (Shah 2017)  721.0x, Cervical spondylosis  721.1x, Cervical spondylosis with myelopathy  722.0x, Displacement of cervical intervertebral disc without myelopathy  722.31, Schmorl’s nodes, thoracic region  722.71, Intervertebral disc disorder with myelopathy, cervical region  722.81, Postlaminectomy syndrome, cervical region  722.91, Other and unspecified disc disorder, cervical region  723.XX, Other disorders of cervical region  839.0, Dislocation; cervical vertebra, closed  839.1, Dislocation; cervical vertebra, open  847.0 Sprain of neck  (Shah 2017) | M05 – M14 (excluding M08 and M09), M15-M25, M30-M36, M40-M53 (excluding M51.3) (ICD Manual) | M05 – M14 (excluding M08 and M09), Seropositive rheumatoid arthritis, other rheumatoid arthritis, psoriatic and enteropathic arthropathies, gout, other crystal arthropathies, other specific arthropathies, other arthritis, arthropathies in other diseases classified elsewhere  M15-M25 (M15-M19 Arthrosis and M20-M25 Other joint disorders): Polyarthrosis; coxarthrosis; gonarthrosis; arthrosis of first carpometacarpal joint; other arthrosis; acquired deformities of fingers and toes; other acquired deformities of limbs; disorders of patella; internal derangement of knee; other specific joint derangement; other joint disorders; not elsewhere classified  M30-M36 (Systemic connective tissue disorders): Polyarteritis nodosa and related conditions; other necrotizing vasculopathies; systemic lupus erythematosus; dermatopolymyositis, systemic sclerosis, other systemic involvement of connective tissue, systemic disorders of connective tissue in diseases classified elsewhere  M40-M53 (excluding M51.3): Kyphosis and lordosis; scoliosis; spinal osteochondrosis; other deforming dorsopathies; ankylosing spondylitis; other spondylopathies; spondylosis; other spondylopathies; spondylopathies in diseases classified elsewhere; cervial disc disorders; other intervertebral disc disorders; other dorsopathies; not elsewhere classified |
| History of fibromyalgia | 729.1x (Shah 2017) | Myalgia with myositis NOS | M79.7 (ICD Manual) | M79.7 Fibromyalgia |
| History of neuropathic pain | 357, 337.0, 356.0, 356.2, 356.4, 356.9, 357.2, 357.3, 531.3, 723.4, 727.2 (Shah 2017) | 357 Inflammatory and toxic neuropathy  337.0, Idiopathic peripheral autonomic neuropathy  356.0, Hereditary peripheral neuropathy  356.2, Hereditary sensory neuropathy  356.4, Idiopathic progressive polyneuropathy  356.9, Unspecified hereditary and idiopathic peripheral neuropathy  357.2, Polyneuropathy in diabetes  357.3, Polyneuropathy in malignant disease  531.3, Acute gastric ulcer; without mention of hemorrhage or perforation  723.4, Brachial neuritis or radiculitis NOS  727.2 Specific bursitides often of occupational origin | G62.8, G62.9, G63.2, G63.3-G63.8, G90.0, G99.0, K25.4-K25.9, K26.4-K26.9, K27.4-K27.9, K28.4-K28.9, M54.1 (ICD Manual) | G62.8, Other specified polyneuropathies  G62.9, Polyneuropathy, unspecified  G63.2, Diabetic polyneuropathy  G63.3-G63.8, Polyneuropathy in other endocrine and metabolic diseases; polyneuropathy in nutritional deficiency; polyneuropathy in systemic connective tissue disorders; polyneuropathy in other musculoskeletal disorders; polyneuropathy in other diseases classified elsewhere.  G90.0, Idiopathic peripheral autonomic neuropathy  G99.0, Other disorders of nervous system in diseases classified elsewhere  K25.4-K25.9, Gastric ulcer, chronic or unspecified with haemorrhage; gastric ulcer, chronic or unspecified with perforation; gastric ulcer, chronic or unspecified with both haemorrhage and perforation; gastric ulcer, chronic without haemorrhage or perforation; gastric ulcer, unspecified as acute or chronic, without haemorrhage or perforation  K26.4-K26.9, Duodenal ulcer, chronic or unspecified with haemorrhage; duodenal ulcer, chronic or unspecified with perforation; duodenal ulcer, chronic or unspecified with both haemorrhage and perforation; duodenal ulcer, chronic without haemorrhage or perforation; duodenal ulcer, unspecified as acute or chronic, without haemorrhage or perforation  K27.4-K27.9, Peptic ulcer, chronic or unspecified with haemorrhage; peptic ulcer, chronic or unspecified with perforation; peptic ulcer, chronic or unspecified with both haemorrhage and perforation; peptic ulcer, chronic without haemorrhage or perforation; peptic ulcer, unspecified as acute or chronic, without haemorrhage or perforation  K28.4-K28.9, Gastrojejunal ulcer, chronic or unspecified with haemorrhage; gastrojejunal ulcer, chronic or unspecified with perforation; gastrojejunal ulcer, chronic or unspecified with both haemorrhage and perforation; gastrojejunal ulcer, chronic without haemorrhage or perforation; gastrojejunal ulcer, unspecified as acute or chronic, without haemorrhage or perforation  M54.1 Radiculopathy |
| History of cancer | All ICD-9-CM malignant neoplasm codes except non-melanoma skin cancer | - | All ICD-10-CA malignant neoplasm codes except non-melanoma skin cancer | - |

Himelhoch S, Weller WE, Wu AW, Anderson GF, Cooper LA. Chronic medical illness, depression, and use of acute medical services among Medicare beneficiaries. Med Care. 2004;42(6):512-21.

Shah A, Hayes CJ, Martin BC. Characteristics of Initial Prescription Episodes and Likelihood of Long-Term Opioid Use - United States, 2006-2015. MMWR Morb Mortal Wkly Rep. 2017;66(10):265-9.

Health Quality Ontario (2019). Low Back Pain: Care for Adults with Low Back Pain. Health Quality Ontario. Toronto, ON: Queen’s Printer for Ontario; 2019. .

Martens P, Nickel N, Forget E, Lix L, Turner D, Prior H, et al. The Cost of Smoking: A Manitoba Study. Winnipeg, MB. Manitoba Centre for Health Policy; 2015.

MCHP Manitoba Centre for Health Policy **(**<http://mchpappserv.cpe.umanitoba.ca/reference//Candata_web_final.pdf#Page=96)> Accessed: May 28 2019; HQ Ontario Health Quality Ontario

**Appendix 6: Results of secondary outcome analysis**

**Table 1: Unadjusted and adjusted odds ratios (with 95% CI) for filling strong opioids and Tramadol based on setting and specialty of procedure provider among opioid naive patients who had surgical or emergency care and filled opioid prescriptions between April 26th 2017 and March 31st 2019 in community pharmacies in Nova Scotia, Canada (n= 36,716).**

|  |  | Strong opioid  (n setting=36,716; n specialty=26,445) | | | Tramadol  (n setting=36,716; n specialty=26,445) | | |
| --- | --- | --- | --- | --- | --- | --- | --- |
|  |  | **n (row %)** | **OR**  **(95% CI)** | **aOR**  **(95% CI)** | **n (row %)** | **OR**  **(95% CI)** | **aOR**  **(95% CI)** |
| Setting | *Surgical care*  *(n=22,773 )* | **15,686 (68. 9)** | Ref | Ref | **1,605 (7.05)** | Ref | Ref |
|  | *Emergency care*  *(n=10,271)* | **5,321 (51.8)** | 0.48  (0.46 – 0.51) | 0.52  (0.50 – 0..55) | **1,355 (13.2)** | 2.00  (1.86 – 2.16) | 1.79  (1.66 – 1.94) |
|  | *Emergency plus surgical care (n=3,672 )* | **2,914 (79.4)** | 1.74  (1.60 – 1.89) | 1.76  (1.62 – 1.92) | **153 (4.17)** | 0.57  (0.48 – 0.68) | 0.57  (0.48 – 0.68) |
| Billing specialty of procedure provider, for all subjects who had procedures | *General Surgery*  *(n=4,041 )* | **2,615 (64.7)** | Ref | Ref | **520 (12.9)** | Ref | Ref |
|  | *Orthopedic surgery (n=4,302)* | **3,203 (74.5)** | 1.59  (1.45 – 1.75) | 1.04  (0.94 – 1.16) | **48 (1.12)** | 0.08  (0.06 – 0.10) | 0.10  (0.08 – 0.14) |
|  | *Obstetrics and Gynecology*  *(n= 2,709)* | **2,282 (84.2)** | 2.91  (2.58 – 3.29) | 2.35  (2.06 – 2.67) | **41 (1.51)** | 0.10  (0.08 – 0.14) | 0.12  (0.09 – 0.17) |
|  | *Plastic Surgery*  *(n= 1,508)* | **618 (41.0)** | 0.38  (0.33 – 0.43) | 0.29  (0.25 – 0.33) | **274 (18.2)** | 1.50  (1.28 – 1.76) | 1.73  (1.46 – 2.04) |
|  | *Otolaryngology*  *(n=1,264)* | **481 (38.1)** | 0.33  (0.29 – 0.38) | 0.35  (0.30 – 0.40) | **311 (24.6)** | 2.21  (1.89 – 2.59) | 2.33  (1.98 – 2.73) |
|  | *Urology*  *(n= 1,208)* | **732 (60.6)** | 0.84  (0.73 – 0.96) | 0.79  (0.69 – 0.91) | **101 (8.36)** | 0.59  (0.46 – 0.76) | 0.64  (0.51 – 0.80) |
|  | *Other Surgical Specialties (n=898)* | **653 (72.7)** | 1.45  (1.24 – 1.71) | 0.93  (0.79 – 1.10) | **72 (8.02)** | 0.59  (0.46 – 0.76) | 0.75  (0.57 – 0.98) |
|  | *General practice (n=9,156 )* | **6,971 (76.1)** | 1.74  (1.60 – 1.88) | 1.31  (1.20 – 1.43) | **318 (3.47)** | 0.24  (0.21 – 0.28) | 0.29  (0.24 – 0.33) |
|  | *Non-surgical non general practice specialties (n=299)* | **128 (42.8)** | 0.41  (0.32 – 0.52) | 0.48  (0.37 – 0.61) | **37 (12.4)** | 0.96  (0.67 – 1.37) | 0.92  (0.64 – 1.32) |
|  | *Unknown**  *(n=1,060)* | **917 (86.5)** | 3.50  (2.90 – 4.22) | 2.88  (2.38 – 3.49) | **36 (3.40)** | 0.24  (0.17 – 0.34) | 0.27  (0.19 – 0.38) |

MME Morphine Milligram Equivalent; OR odds ratio; aOR adjusted odds ratio; CI confidence interval

* Billing specialty of procedure provider not available (CCI code)

Strong opioids: Hydromorphone, oxycodone, morphine, fentanyl; Weak opioids: Codeine, tramadol.

Undergoing procedures identified from MSI Physicians’ Billings using Canadian Classification of Procedures (CCP) codes and CIHI DAD using the Canadian Classification of Health Interventions (CCI) codes after linkage to Drug Information System Database (DIS)

Emergency Department visits identified from NACRS database after linkage to DIS

Billing specialty of procedure provider identified from MSI Physicians’ Billings Database

Covariates included in the adjusted models: patient age (continuous, in years) and sex (binary, female or male) on the day of opioid filling, binary variables (yes vs. no) measuring 12-month history of the following conditions: depression, anxiety, alcohol abuse, drug abuse, tobacco abuse, low back pain, headache, arthritis, fibromyalgia, neuropathic pain, and cancer (any cancer except non-melanoma skin cancer). In the model for specialty of procedure provider, we also adjusted for procedure type (major surgery; minor surgery; fracture, dislocation, or cast; bone grafting; obstetrics; and other, which included procedures that were captured in DAD database but not MSI).

**Appendix 7: Results of sensitivity analyses**

**Table 1: Summary of sensitivity analyses results for the association between setting of care, specialty of procedure provider and prescriptions > 7 days’ supply. Adjusted odds ratios (with 95% CI) presented (n=36,716 unless otherwise indicated).**

|  |  | Days’ Supply >7 days  (unless otherwise indicated: n setting=36,716; n specialty=26,445) | | | | | |
| --- | --- | --- | --- | --- | --- | --- | --- |
|  |  | **Adjusted OR (95% CI)** | | | | | |
|  |  | **Main** | **No history of cancer diagnosis in past 12-m (n= 31,348) ^a^** | **ED visit only eligible if ≤ 2 days from fill**  **(n= 32,191) ^b^** | **Alternative definition provider specialty ^c^** | **Outcome defined > 3 days** ^d^ | **Outcome defined > 14 days** ^d^ |
| Setting | *Surgical care* | **Ref** | Ref | Ref | - | Ref | Ref |
|  | *Emergency care* | **2.13 (1.98 – 2.29)** | 1.99 (1.84 – 2.16) | 1.29 (1.18 – 1.41) | - | 1.43  (1.36 – 1.50) | 3.55  (3.13 – 4.02) |
|  | *Emergency plus surgical care* | **0.86 (0.75 – 0.99)** | 0.81 (0.70 – 0.94) | 0.62 (0.48 – 0.79) | - | 0.75  (0.70 – 0.81) | 1.22  (0.96 – 1.55) |
| Billing specialty of procedure provider | *General Surgery* | **Ref** | Ref | - | Ref | Ref | Ref |
|  | *Orthopedic surgery* | **1.17 (0.94 – 1.47)** | 1.21 (0.94 – 1.55) | - | 2.52 (2.11 – 3.01) | 1.42  (1.28 – 1.57) | 0.89  (0.57 – 1.38) |
|  | *Obstetrics and Gynecology* | **1.46 (1.13 – 1.89)** | 1.71 (1.29 – 2.26) | - | 1.56 (1.23 – 1.97) | 0.99  (0.88 – 1.11) | 1.26  (0.78 – 2.04) |
|  | *Plastic Surgery* | **1.03 (0.75 – 1.40)** | 0.89 (0.62 – 1.28) | - | 1.24 (0.92 – 1.66) | 0.95  (0.83 – 1.09) | 1.13  (0.64 – 1.99) |
|  | *Otolaryngology* | **4.89 (3.86 – 6.20)** | 5.36 (4.07 – 7.05) | - | 5.62 (4.51 – 6.99) | 3.38  (2.96 – 3.87) | 2.68  (1.72 – 4.18) |
|  | *Urology* | **1.19 (0.86 – 1.65)** | 1.14 (0.75 – 1.74) | - | 1.31 (0.96 – 1.79) | 0.72  (0.62 – 0.84) | 1.42  (0.83 – 2.42) |
|  | *Other Surgical Specialties* | **2.52 (1.89 – 3.36)** | 2.88 (2.02 – 4.09) | - | 2.83 (2.16 – 3.72) |  |  |
|  | *General practice* | **2.31**  **(1.91 – 2.79)** | 2.41 (1.93 – 3.00) | - | 4.84 (3.86 – 6.05) | 1.80  (1.65 – 1.96) | 1.26  (0.87 – 1.81) |
|  | *Non-surgical non general practice specialties* | **6.66 (4.87 – 9.11)** | 7.33 (5.16 – 10.4) | - | 8.03 (5.95 – 10.8) | 2.96  (2.30 – 3.80) |  |
|  | *Unknown* | **5.35 (4.19 – 6.85)** | 4.73 (3.50 – 6.40) | - | 5.83 (4.64 – 7.33) | 1.88  (1.63 – 2.17) | 8.19  (5.49 – 12.2) |

MME Morphine Milligram Equivalent; OR odds ratio; aOR adjusted odds ratio; CI confidence interval

* Billing specialty of procedure provider not available (CCI code)

Undergoing procedures identified from MSI Physicians’ Billings using Canadian Classification of Procedures (CCP) codes and CIHI DAD using the Canadian Classification of Health Interventions (CCI) codes after linkage to Drug Information System Database (DIS)

Emergency Department visits identified from NACRS database after linkage to DIS

Billing specialty of procedure provider identified from MSI Physicians’ Billings Database

Covariates included in the adjusted models: patient age (continuous, in years) and sex (binary, female or male) on the day of opioid filling, binary variables (yes vs. no) measuring 12-month history of the following conditions: depression, anxiety, alcohol abuse, drug abuse, tobacco abuse, low back pain, headache, arthritis, fibromyalgia, neuropathic pain, and cancer (any cancer except non-melanoma skin cancer) – codes in Appendix 5. In the model for specialty of procedure provider, we also adjusted for procedure type (major surgery; minor surgery; fracture, dislocation, or cast; bone grafting; obstetrics; and other, which included procedures that were captured in DAD database but not MSI).

**^a^** In this analysis, we excluded 5,368 subjects with a history of cancer in the past 12 months.

**^b^** Here we considered prescriptions to be related to an ED visit only if they were filled on or up to two days from the visit. This excluded 4,748 subjects.

**^c^** In this analysis, we recategorized 7,594 subjects whose procedures were billed by general practice to the second listed surgical specialty of their procedure provider (n= 2,159 moved to general surgery, n= 4,783 moved to orthopedic surgery, and n= 652 moved to obstetrics and gynecology)

^d^ In this sensitivity analysis, we set the threshold for days’ supply at 3 days and 14 days.

**Table 2: Summary of results from sensitivity analyses for the association between setting of care, specialty of procedure provider and prescriptions ≥90MME/day. Adjusted odds ratios (with 95% CI) presented (n=36,716 unless otherwise indicated).**

|  |  | Dose ≥ 90MME/day  (unless otherwise indicated: n setting=36,716; n specialty=26,445) | | | | |
| --- | --- | --- | --- | --- | --- | --- |
|  |  | **Adjusted OR (95% CI)** | | | | |
|  |  | **Main** | **No history of cancer diagnosis in past 12-m (n= 31,348) ^a^** | **ED visit only eligible if ≤ 2 days from fill (n= 32,191) ^b^** | **Alternative definition provider specialty ^c^** | **Outcome defined as ≥50 MME/d** ^d^ |
| Setting | *Surgical care* | **Ref** | Ref | Ref | - | Ref |
|  | *Emergency care* | **0.34**  **(0.31 – 0.37)** | 0.33 (0.31 – 0.36) | 0.38  (0.35 – 0.41) | - | 0.34  (0.33 – 0.36) |
|  | *Emergency plus surgical care* | **1.07**  **(0.99– 1.16)** | 1.08 (1.00 – 1.18) | 0.87 (0.76 – 0.99) | - | 1.10  (1.03 – 1.19) |
| Billing specialty of procedure provider | *General Surgery* | **Ref** | Ref | - | Ref | Ref |
|  | *Orthopedic surgery* | **2.92**  **(2.58 – 3.30)** | 2.82 (2.46 – 3.23) | - | 3.93 (3.54 – 4.34) | 1.96  (1.77 – 2.17) |
|  | *Obstetrics and Gynecology* | **1.94**  **(1.68 – 2.22)** | 2.20 (1.88 – 2.57) | - | 2.05 (1.81 – 2.31) | 1.42  (1.28 – 1.58) |
|  | *Plastic Surgery* | **0.74**  **(0.62 – 0.89)** | 0.70 (0.57 – 0.86) | - | 0.85 (0.71 – 1.01) | 0.49  (0.44 – 0.56) |
|  | *Otolaryngology* | **0.63**  **(0.50 – 0.79)** | 0.62 (0.48 – 0.82) | - | 0.61 (0.49 – 0.77) | 0.76  (0.67 – 0.87) |
|  | *Urology* | **0.68**  **(0.54 – 0.85)** | 0.56 (0.41 – 0.76) | - | 0.67 (0.54 – 0.82) | 0.72  (0.63 – 0.83) |
|  | *Other Surgical Specialties* | **1.52**  **(1.26 – 1.84)** | 0.89 (0.68 – 1.17) | - | 1.62 (1.35 – 1.93) | 1.22  (1.05 – 1.42) |
|  | *General practice* | **2.45**  **(2.20 – 2.74)** | 2.46 (2.18 – 2.79) | - | 1.41 (1.18 – 1.68) | 1.69  (1.56 – 1.83) |
|  | *Non-surgical non general practice specialties* | **0.74**  **(0.48 – 1.13)** | 0.54 (0.31 – 0.93) | - | 0.7 (0.50 – 1.17) | 0.43  (0.33 – 0.57) |
|  | *Unknown* | **3.06**  **(2.60 – 3.60)** | 3.46 (2.87 – 4.17) | - | 3.09 (2.65 – 3.59) | 1.48  (1.29 – 1.71) |

MME Morphine Milligram Equivalent; OR odds ratio; aOR adjusted odds ratio; CI confidence interval

* Billing specialty of procedure provider not available (CCI code)

Undergoing procedures identified from MSI Physicians’ Billings using Canadian Classification of Procedures (CCP) codes and CIHI DAD using the Canadian Classification of Health Interventions (CCI) codes after linkage to Drug Information System Database (DIS)

Emergency Department visits identified from NACRS database after linkage to DIS

Billing specialty of procedure provider identified from MSI Physicians’ Billings Database

Covariates included in the adjusted models: patient age (continuous, in years) and sex (binary, female or male) on the day of opioid filling, binary variables (yes vs. no) measuring 12-month history of the following conditions: depression, anxiety, alcohol abuse, drug abuse, tobacco abuse, low back pain, headache, arthritis, fibromyalgia, neuropathic pain, and cancer (any cancer except non-melanoma skin cancer) – codes in Appendix 5. In the model for specialty of procedure provider, we also adjusted for procedure type (major surgery; minor surgery; fracture, dislocation, or cast; bone grafting; obstetrics; and other, which included procedures that were captured in DAD database but not MSI).

**^a^** In this analysis, we excluded 5,368 subjects with a history of cancer in the past 12 months.

**^b^** Here we considered prescriptions to be related to an ED visit only if they were filled on or up to two days from the visit. This excluded 4,748 subjects.

**^c^** In this analysis, we recategorized 7,594 subjects whose procedures were billed by general practice to the second listed surgical specialty of their procedure provider (n= 2,159 moved to general surgery, n= 4,783 moved to orthopedic surgery, and n= 652 moved to obstetrics and gynecology)

^d^ In this sensitivity analysis, we set the threshold for dose at 50 MME/day.

**Table 3: Summary of sensitivity analyses results for the association between setting of care, specialty of procedure provider and long-acting opioid prescriptions. Adjusted odds ratios (with 95% CI) presented (n=36,716 unless otherwise indicated).**

|  |  | Long-acting  (unless otherwise indicated: n setting=36,716; n specialty=26,445) | | | |
| --- | --- | --- | --- | --- | --- |
|  |  | **Adjusted OR (95% CI)** | | | |
|  |  | **Main** | **No history of cancer diagnosis in past 12-m (n= 31,348) ^a^** | **ED visit only eligible if ≤ 2 days from fill (n= 32,191) ^b^** | **Alternative definition provider specialty ^c^** |
| Setting | *Surgical care* | **Ref** | Ref | Ref | - |
|  | *Emergency care* | **1.36**  **(1.04 – 1.77)** | 1.20 (0.89 – 1.62) | 0.72 (0.51 - 1.02) | - |
|  | *Emergency plus surgical care* | **0.44**  **(0.23 – 0.82)** | 0.28 (0.12 – 0.63) | 0.29 (0.09 – 0.93) | - |
| Billing specialty of procedure provider | *General Surgery* | **Ref** | Ref | - | Ref |
|  | *Orthopedic surgery* | **0.17**  **(0.06 – 0.45)** | 0.14 (0.05 – 0.42) | - | 0.08 (0.04 – 0.15) |
|  | *Obstetrics and Gynecology* | **-** | - | - | - |
|  | *Plastic Surgery* | **0.16**  **(0.04 – 0.67)** | 0.20 (0.05 – 0.84) | - | 0.09 (0.02 – 0.35) |
|  | *Otolaryngology* | **0.07**  **(0.01 – 0.50)** | - | - | 0.05 (0.01 – 0.35) |
|  | *Urology* | **0.90**  **(0.48 – 1.70)** | 1.10 (0.54 – 2.23) | - | 0.65 (0.36 – 1.17) |
|  | *Other Surgical Specialties* | **0.09**  **(0.01 – 0.70)** | 0.16 (0.02 – 1.20) | - | 0.06 (0.01 – 0.43) |
|  | *General practice* | **0.89**  **(0.59 – 1.36)** | 0.84 (0.53 – 1.33) | - | 0.28 (0.11 – 0.72) |
|  | *Non-surgical non general practice specialties* | **2.12**  **(0.98 – 4.60)** | 2.34 (1.02 – 5.29) | - | 1.31 (0.62 – 2.76) |
|  | *Unknown* | **2.10**  **(1.24 – 3.55)** | 0.58 (0.23 – 1.51) | - | 1.41 (0.87 – 2.27) |

MME Morphine Milligram Equivalent; OR odds ratio; aOR adjusted odds ratio; CI confidence interval

* Billing specialty of procedure provider not available (CCI code)

Undergoing procedures identified from MSI Physicians’ Billings using Canadian Classification of Procedures (CCP) codes and CIHI DAD using the Canadian Classification of Health Interventions (CCI) codes after linkage to Drug Information System Database (DIS)

Emergency Department visits identified from NACRS database after linkage to DIS

Billing specialty of procedure provider identified from MSI Physicians’ Billings Database

Covariates included in the adjusted models: patient age (continuous, in years) and sex (binary, female or male) on the day of opioid filling, binary variables (yes vs. no) measuring 12-month history of the following conditions: depression, anxiety, alcohol abuse, drug abuse, tobacco abuse, low back pain, headache, arthritis, fibromyalgia, neuropathic pain, and cancer (any cancer except non-melanoma skin cancer) – codes in Appendix 5. In the model for specialty of procedure provider, we also adjusted for procedure type (major surgery; minor surgery; fracture, dislocation, or cast; bone grafting; obstetrics; and other, which included procedures that were captured in DAD database but not MSI).

**^a^** In this analysis, we excluded 5,368 subjects with a history of cancer in the past 12 months.

**^b^** Here we considered prescriptions to be related to an ED visit only if they were filled on or up to two days from the visit. This excluded 4,748 subjects.

**^c^** In this analysis, we recategorized 7,594 subjects whose procedures were billed by general practice to the second listed surgical specialty of their procedure provider (n= 2,159 moved to general surgery, n= 4,783 moved to orthopedic surgery, and n= 652 moved to obstetrics and gynecology)
